# Supplementary figures and images for: An exploration of the emotional response among nurses in Bermuda, during the Covid-19 pandemic
Source: PLoS One. 2024 Sep 17;19(9):e0279792. doi: 10.1371/journal.pone.0279792 (PMC11407671; doi:10.1371/journal.pone.0279792)

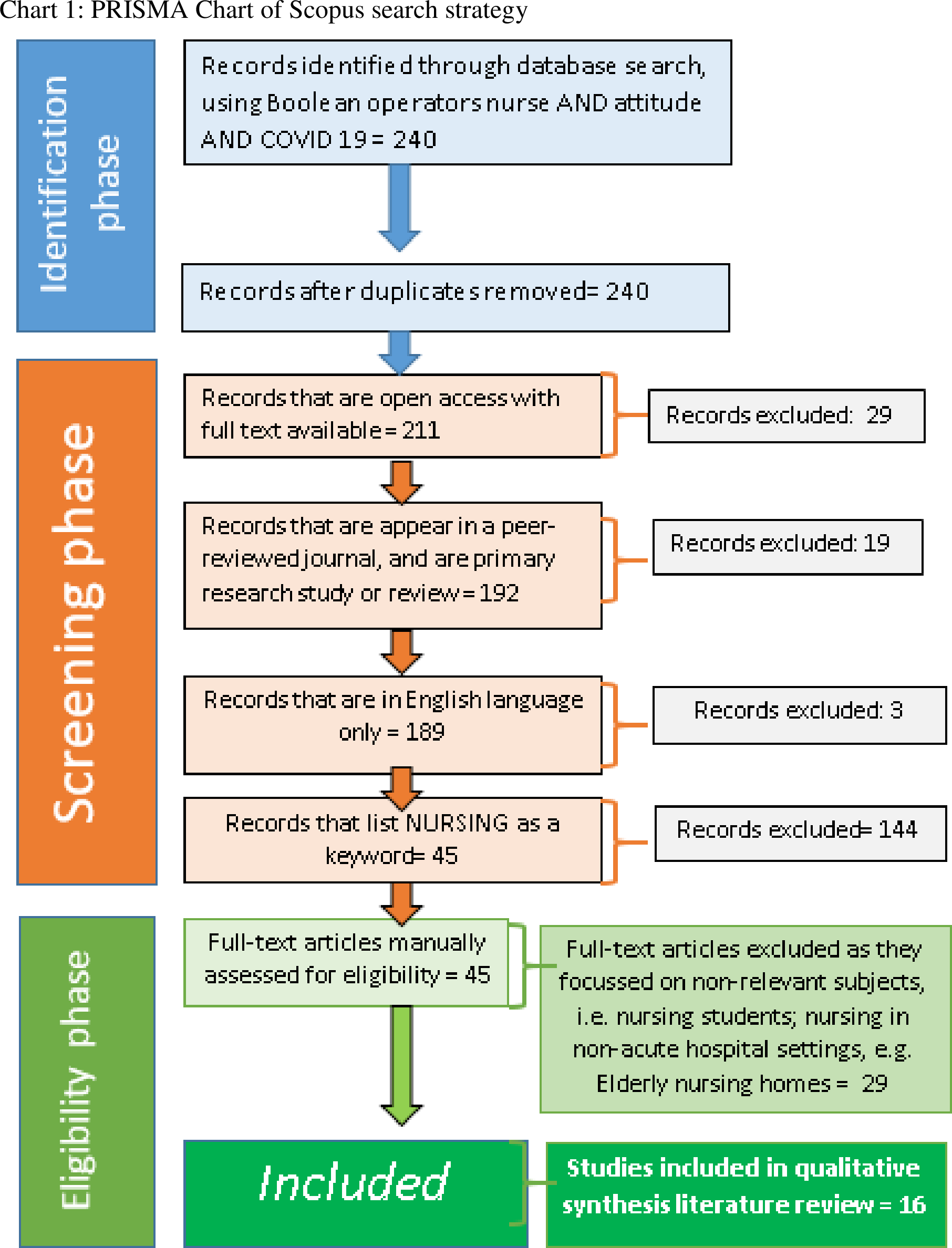

Supplement: S1 File — (TIF) [file pone.0279792.s001.tif]

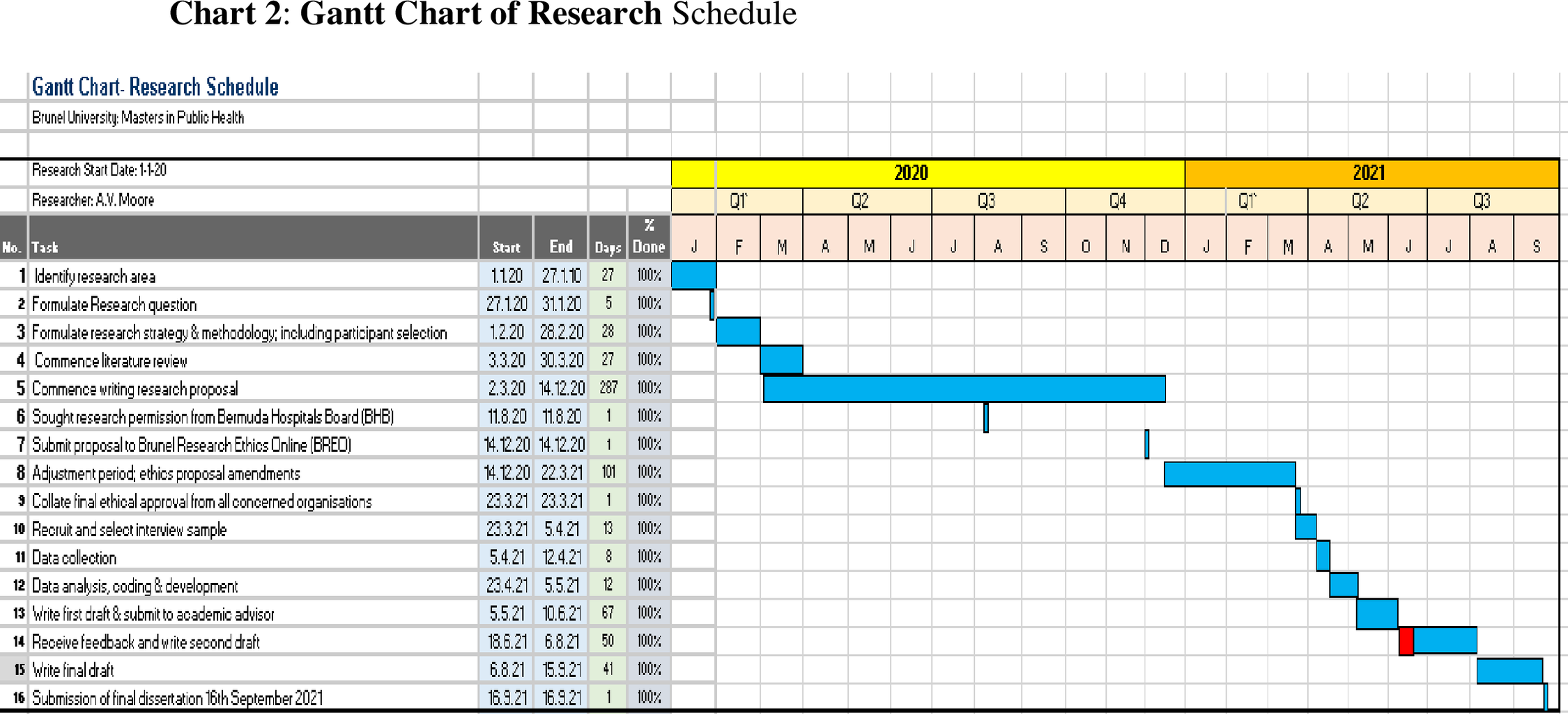

Supplement: S2 File — (TIF) [file pone.0279792.s002.tif]

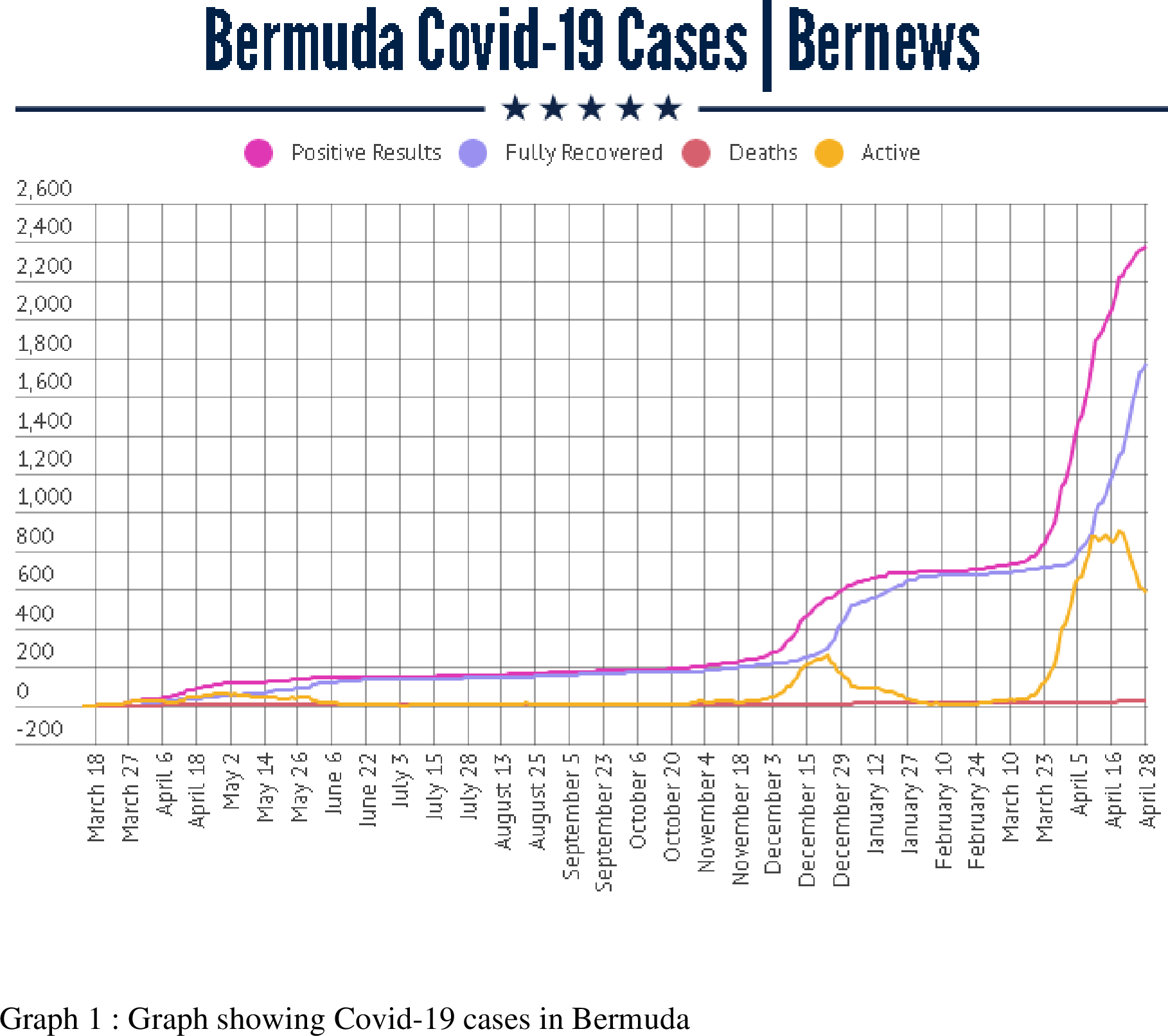

Supplement: S3 File — (TIF) [file pone.0279792.s003.tif]

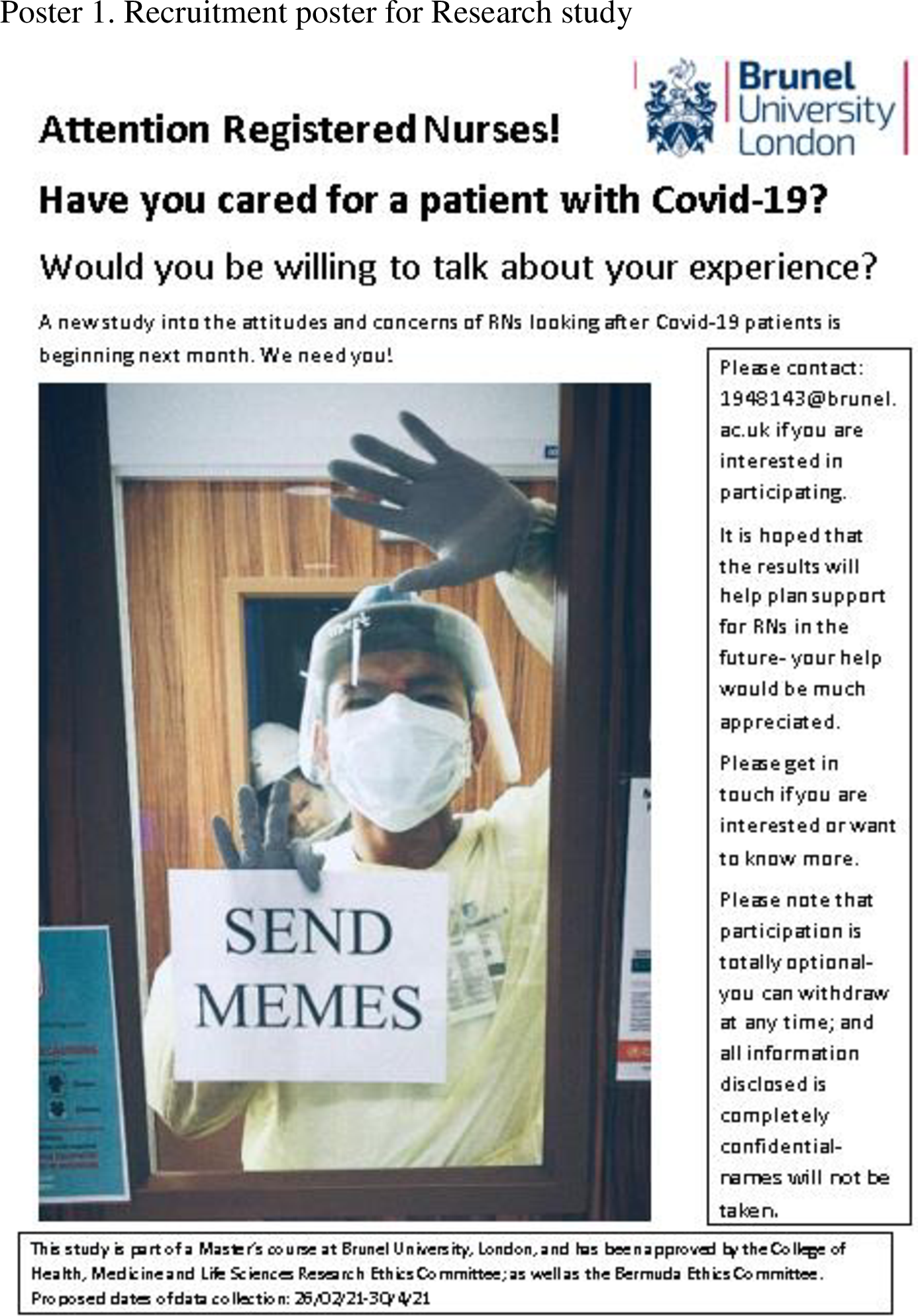

Supplement: S4 File — (TIF) [file pone.0279792.s004.tif]

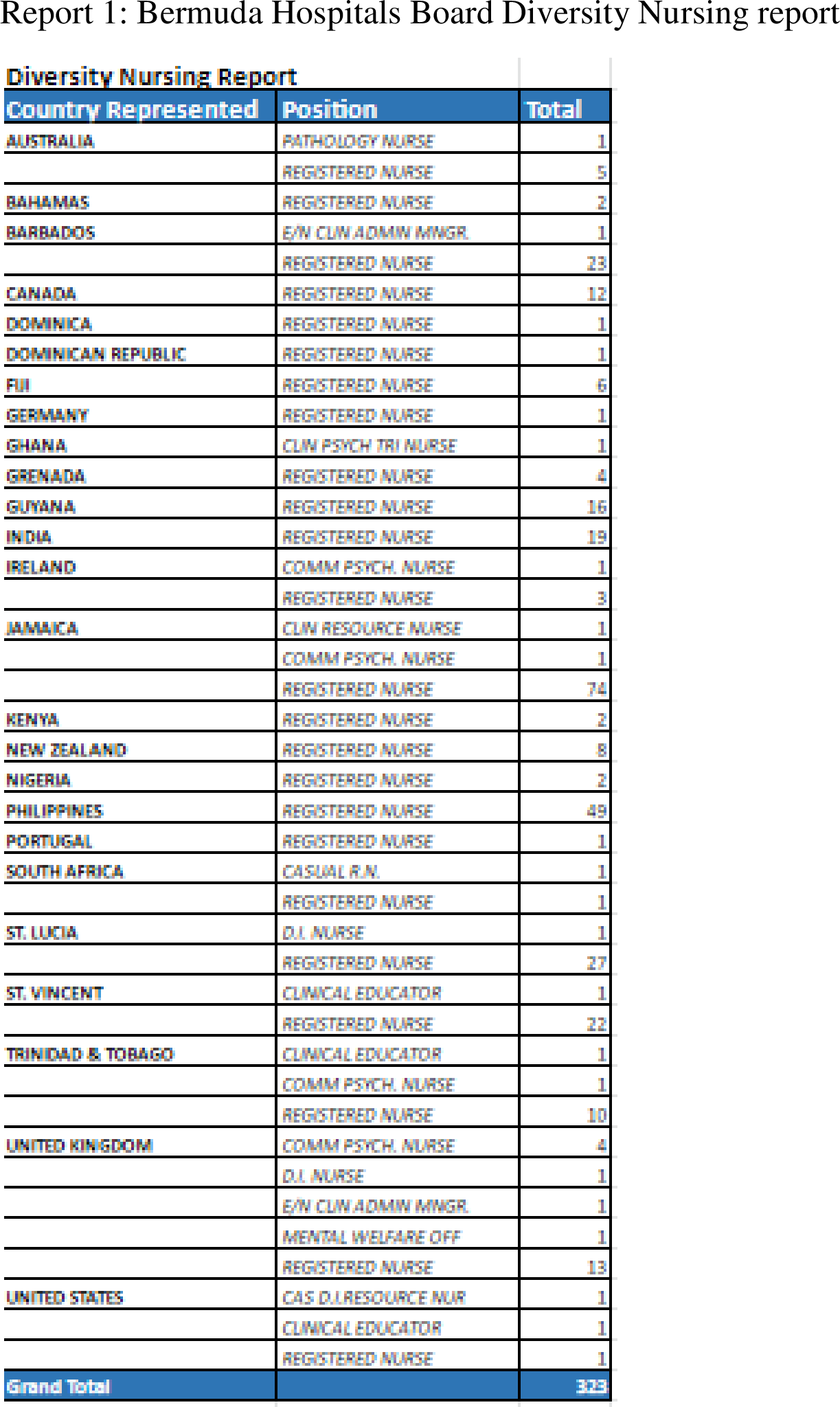

Supplement: S5 File — (TIF) [file pone.0279792.s005.tif]
