# Supplementary material for: An exploration of the emotional response among nurses in Bermuda, during the Covid-19 pandemic
Source: PLoS One. 2024 Sep 17;19(9):e0279792. doi: 10.1371/journal.pone.0279792 (PMC11407671; doi:10.1371/journal.pone.0279792)
Supplement: S1 Appendix — (DOCX) [file pone.0279792.s006.docx]

**Appendices:**

Appendix A. Literature review table

| Study title, DOI | Type of research/data collection tools used | Population | Themes identified |
| --- | --- | --- | --- |
| 1. Impact of the coronavirus disease 2019 pandemic on critical care healthcare workers' depression, anxiety, and stress levels   [https://doi.org/10.1016/j.aucc.2020.12.004](file:///C:\h) | Survey. Anonymous web-based study, of HCW in Australia And New Zealand. Utilised the Depression, Anxiety, and Stress Scale-21 (DASS-21). | 3770 complete responses, 3039 (80.6%) were from Australia.  A total of 2871 respondents (76.2%) were women; the median age was 41 years.  Nurses made up 2269 (60.2%) of respondents, with most (2029 [53.8%]) working in intensive care units. | Between 22 and 29% of respondents reported moderate to extremely severe depression, anxiety, and stress symptoms, with women reporting higher scores than men. Although female gender appears to play a role, modifiable factors also contribute to psychological burden and should be studied further. |
| 1. Exploring nurses' perception of taking care of patients with coronavirus disease (COVID-19): A qualitative study [12]   <https://doi.org/10.1002/nop2.616> | Qualitative. The data were collected through telephone interviews and analysed based on the Lundman and Graneheim's approach. | 13 nurses who were taking care of patients with COVID-19, recruited from  Lorestan University of Medical Sciences, Khorramabad Iran | Care erosion, nursing professional growth and necessities. Relevance to clinical practice: Improve the work situation of the nurses during care of COVID-19 patients, which can directly or indirectly improve the quality of care of these patients. |
| Nurses’ experiences regarding shift patterns in isolation wards during the COVID-19 pandemic in China: A qualitative study  <https://doi-org.ezproxy.brunel.ac.uk/10.1111/jocn.15464> | Qualitative: phenomenological; Methods: Semi-structured interviews were used based on the phenomenological research method; | 14 nurses from Chinese hospitals | Focussed on shift organisation; recognising need for necessary to arrange shift patterns scientifically and allocate workforce rationally to optimise nursing workforce allocation, reduce nurses’ workload, improve nursing quality and promote physical and mental health among nurses during the COVID-19 pandemic |
| 1. Psychological impact of COVID-19 outbreak on frontline nurses: A cross-sectional survey study [27]   <https://doi-org.ezproxy.brunel.ac.uk/10.1111/jocn.15454>  [27] Psychological impact of COVID‐19 outbreak on frontline nurses: A cross‐sectional survey study. *Journal of clinical nursing*, *29*(21-22), 4217-4226. | Cross-sectional study. Methods: Frontline nurses complete an online survey by convenience sampling, and the survey included six main sections: the General Health Questionnaire, the Perceived Social Support Scale, the Simplified Coping Style Scale, the Impact of Event Scale-Revised, socio-demographic, occupation and work history. | 263 frontline nurses from Guangdong Province, China | Concern for family, being treated differently, negative coping style and COVID-19-related stress symptom were positive related to psychological distress. Perceived more social support and effective precautionary measures were negatively associated with psychological distress |
| 1. Implications for COVID-19: A systematic review of nurses’ experiences of working in acute care hospital settings during a respiratory pandemic [10]   [https://doi.org/10.1016/j.ijnurstu.2020.103637](file:///C:\h) | Systematic review of literature:  A structured search using CINAHL, MEDLINE, EMBASE, PubMed, Google Scholar, Cochrane Library, MedNar, ProQuest and Index to Theses was conducted. Review methods: | Thirteen qualitative studies were included in the review. The experiences of 348 nurses included. | Emphasis on supportive care; dedication. Nurses’ require Governments, policy makers and nursing groups to actively engage in supporting nurses, both during and following a pandemic or epidemic. Without this, nurses are likely to experience substantial psychological issues that can lead to burnout and loss from the nursing workforce. |
| 1. Factors associated with nurses' willingness to participate in care of patients with COVID‐19: A survey in China [42] <https://doi-org.ezproxy.brunel.ac.uk/10.1111/jonm.13126> | This cross-sectional study used a convenience sampling method that included a demographics questionnaire and the Nurses' Perceived Professional Benefits Questionnaire. | The survey was distributed to 1,787 nurses from 36 hospitals in China. Results: In total, 1,176 questionnaires were usable for this research | The vast majority of nurses were willing to participate in care of patients with COVID-19 in China. Surgical nurses and nurses with positive professional perceptions are more likely to be willing to participate in treatment. raising the awareness of infectious diseases and increased pre-disaster training during infectious diseases is critical |
| 1. Caring for the caregiver during COVID-19 outbreak: Does inclusive leadership improve psychological safety and curb psychological distress? A cross-sectional study [42] [https://doi.org/10.1016/j.ijnurstu.2020.103725](https://doi-org.ezproxy.brunel.ac.uk/10.1016/j.ijnurstu.2020.103725) | Design: Cross-Sectional Study with Temporal Separation Settings and Participants: Online questtionare | The researchers recruited 451 on-duty registered nurses from 5 hospitals providing patient care during the highly infectious phase of COVID-19 in January 2020 in Wuhan city. | Recurring or prolonged experiences of stress and anxiety at the workplace, without a mechanism to counter such effects, can culminate into psychological distress. Inclusive leadership style can serve as such a mechanism to curb psychological distress for healthcare workers by creating a psychologically safe environment |
| 1. Psychological effects of nurses and midwives due to COVID-19 outbreak: The case of Turkey [1] [https://doi.org/10.1016/j.apnu.2020.07.011](https://doi-org.ezproxy.brunel.ac.uk/10.1016/j.apnu.2020.07.011) | Questionnaire: Personal Information Form, State-Trait Anxiety Inventory and Intolerance of Uncertainty Scale were used as data collection tools | 758 total HCWs:  56.9% of nurses and 43.1% of midwives (Recruited from one HC facility in Turkey) | Nurses and midwives are more likely to suffer from anxiety during Covid outbreak .   It was determined that there was a difference between scale scores and difficulties in work, family and private life due to COVID-19 |
| 1. Experiences of clinical first-line nurses treating patients with COVID-19: A qualitative study   [24]   <https://doi-org.ezproxy.brunel.ac.uk/10.1111/jonm.13095> | Qualitative interviews | Thirty nurses were selected for the study using a purposive sampling method. Data were collected by semi-structured interviews and analysed using content analysis. | Negative psychological experiences of clinical first-line work and difficulties faced during hands on work. Identification of need for further support, education, training, counselling. |
| 1. Experiences of front-line nurses combating coronavirus disease-2019 in China: A qualitative analysis [24] 2. <https://doi-org.ezproxy.brunel.ac.uk/10.1111/phn.12768> | Qualitative analysis | Fifteen front-line nurses caring for COVID-19 patients were recruited from two hospitals in Wuhan, China from January 26 to February 5, 2020. Data were collected through semi-structured individual interviews and analyzed using standard qualitative methods. | Facing tremendous new challenges and danger”; “Strong pressure because of fear of infection, exhaustion by heavy workloads and stress of nursing seriously ill COVID-19 patients”; “Strong sense of duty and identity as a healthcare provider”; “Rational understanding of the epidemic’’, need for further training |
| 1. A Comparison of Burnout Frequency Among Oncology Physicians and Nurses Working on the Frontline and Usual Wards During the COVID-19 Epidemic in Wuhan, China [39] [https://doi.org/10.1016/j.jpainsymman.2020.04.008](https://doi-org.ezproxy.brunel.ac.uk/10.1016/j.jpainsymman.2020.04.008) | Questionnaire, 49 questions, 220 medical staff members from the COVID-19 FL and UWs, with a ratio of 1:1. General information, and the Maslach Burnout Inventory—medical personnel, were gathered | 220 nurses and Doctors working in one oncology unit in Wuhan, China, | Staff working on ‘usual wards’ s for uninfected patients, medical staff working on the COVID-19 rather than frontline wards had a lower frequency of burnout. Recognition that it is not just frontline staff affected by pandemic- normal operating systems are put on hold for entire HC system . |
| 1. A study on the psychological needs of nurses caring for patients with coronavirus disease 2019 from the perspective of the existence, relatedness, and growth theory [40] <https://doi.org/10.1016/j.ijnss.2020.04.002> | Qualitative, indepth interviews | 10 nurses working with COvid patients in Wuhan, China. | Existence, relatedness, and growth needs coexist in clinical nurses.- i.e. need to see friends and family face to face as well as have support systems within work environment.  The existence needs were mainly reflected in health and security needs, whereas the relatedness needs consisting mainly of interpersonal needs, humanistic concern needs, and family needs; further, the growth needs consisting mainly of knowledge needs. |
| 1. Coronavirus disease 2019 crisis in Paris: A differential psychological impact between regular intensive care unit staff members and reinforcement workers [2]   [https://doi.org/10.1016/j.aucc.2020.11.005](file:///C:\h) | Self assessment questionnaires. Anxiety and Depression Scale, the Post-traumatic Stress Disorder Checklist for DSM-5, McGill Quality of Life Questionnaire-Revised, and 10-item Connor-Davidson Resilience Scale | Results: Sixty-nine ICU HCWs completed the survey (37 from the team of regular staff members, i.e., from the public health service, and 32 from a reinforcement pool, either from non-ICU public health service or from private healthcare interim employment agencies), from 16-bed neurological ICU at La Pitié-Salpêtrière Hospital in Paris, France, which was converted to a COVID ICU. | Prevalence of anxiety, depression, and post-traumatic stress disorder symptoms was high. Regular staff members appeared to be more at risk from suffering from PTSD-like symptoms than ‘reinforcement’ or additional staff members. |
| 1. Novice nurse's transitioning to emergency nurse during COVID-19 pandemic: A qualitative study [13]   <https://doi-org.ezproxy.brunel.ac.uk/10.1111/jonm.13148> | Qualitative: Semi structured interviews. | Sixteen semi-structured interviews were conducted in sampling at EDs in three different hospitals in Almeria (Andalusia, Spain) | (a) Fears and concerns- i.e. stress; anxiety; infecting a family member  (b) Organisational issues- lack of planning/bed space; over crowding  (c) Support for novice nurses: need improved communication, better support for less experienced colleagues |
| 15Burnout and its influencing factors between frontline nurses and nurses from other wards during the outbreak of Coronavirus Disease-COVID-19-in Iran [17] <https://doi.org/10.17533/udea.iee.v38n2e03> | Cross-sectional study makes comparison between two groups of nurses including frontline (exposure group) and other nurses working in usual wards (non-exposure group) in TorbatHeydariyeh city, Iran. | 245  Nurses; (response rate 92%). According to hospital  policies, frontline nurses as an exposure group  have to work 12 hour-rotating shifts for one week  and have a week off, while other nurses as a non-  exposure group entered in the study have the  same working condition as before | The burnout level in frontline nurses was higher than other nurses, the most important influencing factor was the job stress. |
| 16.Psychological Change Process of Frontline Nurses Caring for Patients with COVID-19 during Its Outbreak [41] <https://doi.org/10.1080/01612840.2020.1752865> | Qualitative descriptive study; semi-stuctured interviews | 23 nurses from Covid wards in Wuhan, China | Stress, anxiety, burnout, emotional exhaustion- transitioning to energy renewal; need for work related support. |

Appendix B. Outline of Interview Questions

**Interview plan sample questions**

**Questions will be used as starting points for general discussion about attitudes to nursing patients with Covid19. As interviews progress, the program is flexible, and will allow for an organic discussion to develop, with plenty of opportunity for participants to speak freely about the issues they found to be pertinent when caring for Covid patients.**

How many patients have you looked after with Covid-19, or possible Covid-19?

How has looking after these patients made you feel?

Has your life changed outside of work because of your status as a nurse? If so, how?

What has helped you during the pandemic so far? Both inside, and outside of work?

Have any changes been made at work to support you while looking after Covid patients?

Would you have liked anything else to be done? Or if there was to be another wave, would you want anything done differently?

Where do you get your information about Covid 19?

Have you heard things about Covid 19 that you don’t believe? Where did you hear them?

How do you feel about the Covid 19 vaccine(s)?

Is there anything else you want to add?

Appendix C. Participant Information Sheet

College of Health, Medicine and Life Sciences

Department of Health Sciences

PARTICIPANT INFORMATION SHEET

**Study title**

Research question: What are the attitudes and concerns of nurses caring for patients with Covid-19 in Bermuda?

**Invitation Paragraph**

You have been invited to participate in this study investigating

nurse attitudes and concerns about caring for patients with Covid-19. Please see

Information below, and get in touch if you want to take part, or have any further

questions. My contact details are at the bottom of the sheet.

**What is the purpose of the study?**

It is hoped that this research will help us to better understand the experience of nurses caring for patients with the virus; in the hope that hospital management will be able to provide tailored support to nurses and other Healthcare professions during this time.

This study is a pre-requisite for the completion on the Masters in Public Health course at Brunel University.

**Why have I been invited to participate?**

You are invited to participate in this study because you are a Registered Nurse working as Bermuda Hospitals Board, and have likely looked after patients with Covid-19. This

study is interested in what you have to say.

**Do I have to take part?**

No. Participation is entirely voluntary. You do not have to take part, and if you decide you would like to participate, you can withdraw at any time with no further questions asked. If you decide to take part, you will be given this information sheet, and will be asked to sign a consent form. Each participant has the right to withdraw at any

time; and you are at liberty to refuse to answer a question or questions without any

consequence at all. If you decide to partcipate, you are still free to withdraw your data, without giving any reason, until 14 days after your interview has taken place.

**What will happen to me if I take part?**

The researcher, Adam Moore, will get in touch to arrange a mutually convenient time to ask some questions about your attitudes to caring for Covid positive patients. This will be over Zoom, Skype, or a phone call. The interview will take 30 minutes at the most. Interviews will be audio taped in order that I can listen back and analyse them afterwards.

**Are there any lifestyle restrictions?**

There are no lifestyle restrictions to this study.

**What are the possible disadvantages and risks of taking part?**

As covid-19 patients are often very sick; it is anticipated that some information that participants wish to share may be upsetting. Please be aware that information shared will be treated with upmost confidence; and not traceable back to an individual. If content shared is highlights risk to patients, the researcher has a duty to inform Quality and Risk.

**What are the possible benefits of taking part?**

This study presents an exciting opportunity for nurses to share how they feel about nursing patients with Covid 19. This pandemic has changed so many things about daily life globally, and is a hot topic, not just in healthcare terms, but also socially, politically, economically. Nurses have been at the forefront of direct patient care during these challenging times, and a more detailed understanding of the pressures they face, and their overall experience dealing with Covid19, will be of great interest to all in the healthcare field, and useful for healthcare planners in the future. This is an opportunity to have your voice heard.

**What if something goes wrong?**

Participants can withdraw at any time. If participants become distressed or upset during interviews, the interview will be terminated. There will be signposting to support services- information on debrief sheet. There will also be a ‘cooling off’ period. After data has been collected, participants may decide that they do not want it included in the study; and may contact the researcher in the 14 days following- any such request will be honoured.

**Will my taking part in this study be kept confidential?**

Yes, data will be completely confidential. Names will not be taken, so there is no way that anything a participant states in an interview could be attributable to them

**Will I be recorded, and how will the recording be used?**

Everything you say will be completely confidential. Individual participants’ names will not

be recorded; there is no way that anything you say will be attributable to you. Signed Consent

forms will be kept in a sealed file, separate to all other data. Recorded data will be analysed by the researcher, and findings will be submitted in the form of a thesis to Brunel University exams board on completion towards the end of 2021.

**What will happen to the results of the research study?**
Results will be coded (for anonymity) and analysed by the researcher before being reported. The results will be written into a research report, the final piece of work for a Masters’ in Public Health, and may later to reported in a scientific journal. In addition, the report will be made available to Bermuda Hospitals Board of Directors. The anonymised research may be shared with other researchers for further analysis; but at no point will uniquely identifiable data be shared. If you choose to take part in this research, you can obtain a copy of the publication from the researcher.

**Who is organising and funding the research?**

Research is organised and funded by Adam Moore, Registered Nurse working on Catlin Lindo Ward, as part of a Masters in Public Health, studying remotely from Brunel University, London.

**What are the indemnity arrangements?**

Brunel University London provides appropriate insurance cover for research which has received ethical approval.

**Who has reviewed the study?**

This study has been reviewed by the College of Health, Medicine and Life Sciences Research Ethics Committee; and also by Bermuda Ethics Committee.

Research Integrity

*Brunel University London is committed to compliance with the Universities UK* [*Research Integrity Concordat*](http://www.universitiesuk.ac.uk/policy-and-analysis/reports/Documents/2012/the-concordat-to-support-research-integrity.pdf)*. You are entitled to expect the highest level of integrity from the researchers during the course of this research*

Contact for further information and complaints:

For general information

Researcher name: Adam Moore.

Email: 1948143@brunel.ac.uk

University Supervisor: Dr.SathyanarayananDoraiswamy

Email: [Sathyanarayanan.Doraiswamy@brunel.ac.uk](mailto:Sathyanarayanan.Doraiswamy@brunel.ac.uk)
