# Supplementary material for: An exploration of the emotional response among nurses in Bermuda, during the Covid-19 pandemic
Source: PLoS One. 2024 Sep 17;19(9):e0279792. doi: 10.1371/journal.pone.0279792 (PMC11407671; doi:10.1371/journal.pone.0279792)
Supplement: Attachment — Submitted filename: PONE-D-22-34212.pdf [file pone.0279792.s007.pdf]

PLOS ONE

An Exploration of the Emotional Response among Nurses in Bermuda, during the Covid-19 Pandemic

--Manuscript Draft--

**Manuscript Number:**	PONE-D-22-34212
**Article Type:**	Research Article
**Full Title:**	An Exploration of the Emotional Response among Nurses in Bermuda, during the Covid-19 Pandemic
**Short Title:**	An Exploration of the Emotional Response among Nurses in Bermuda, during the Covid-19 Pandemic
**Corresponding Author:**	Adam Moore<br>Bermuda Hospitals Board<br>hamilton, BERMUDA
**Keywords:**	covid, covid-19, pandemic, respiratory illness, nurses, emotional response, stress, burnout, isolation, healthcare, nurse, bermuda
**Abstract:**	The Novel Coronavirus Covid-19 has had a huge impact on healthcare systems globally. Nurses spend the most time providing one-to-one care for Covid-19 patients; and are most at risk from contracting the virus (Sethi, 2020). During the course of the Covid-19 pandemic to date; nurses caring for patients within an already undersubscribed workforce have weathered challenging shifts with heavy workloads, and limited equipment (Jackson et al., 2020; International Council of Nurses, 2020). Data from previous respiratory pandemics has shown that nurses suffer from stress and anxiety due to challenges faced inside of work; and stigma in the community, with concern about passing the disease to loved ones exacerbating feelings of isolation (Gao et al., 2020). The Covid-19 pandemic is predicted to bring about further exodus from the workforce; (WHO, 2020) and it is imperative that research be conducted into how to better support nurses, physically and emotionally, so that they avoid burnout, and can continue to provide compassionate care.<br>This paper demonstrates that nurses working in Bermuda are no exception in terms of feeling stress and anxiety at the outset of the pandemic. This thesis reviews international literature; and investigates the emotional responses of nurses in Bermuda, analysing data from interviews with front-line nurses. It is established that confidence in treating Covid-19 patients has grown as a result of developments in research and availability of the vaccine; and workplace camaraderie between nurses provides a valued support network. Nurses express that they could be better supported by clearer communication of pandemic planning as it evolves; and better access to vital equipment to keep themselves and their patients safe. More research is recommended to better inform interventions aimed at providing improved holistic care for nurses: policies to offer counselling and support; easier access to activities that nurses found beneficial; and pandemic preparedness education.
**Order of Authors:**	Adam Moore
	Navami Leena, BDS,MBA,PgCert HE,FHEA,FRSPH,MIHM
**Additional Information:**	
**Question**	**Response**
**Financial Disclosure**<br><br>Enter a financial disclosure statement that describes the sources of funding for the work included in this submission. Review the submission guidelines for detailed requirements. View published research articles from *PLOS ONE* for specific examples.	The author received no specific funding for this work.

This statement is required for submission and **will appear in the published article** if the submission is accepted. Please make sure it is accurate.<br><br>**Unfunded studies**<br>Enter: *The author(s) received no specific funding for this work.*<br><br>**Funded studies**<br>Enter a statement with the following details:<br>• Initials of the authors who received each award<br>• Grant numbers awarded to each author<br>• The full name of each funder<br>• URL of each funder website<br>• Did the sponsors or funders play any role in the study design, data collection and analysis, decision to publish, or preparation of the manuscript?<br>• **NO** - Include this sentence at the end of your statement: *The funders had no role in study design, data collection and analysis, decision to publish, or preparation of the manuscript.*<br>• **YES** - Specify the role(s) played.<br><br>* typeset	
**Competing Interests**<br><br>Use the instructions below to enter a competing interest statement for this submission. On behalf of all authors, disclose any competing interests that could be perceived to bias this work—acknowledging all financial support and any other relevant financial or non-financial competing interests.<br><br>This statement is required for submission and **will appear in the published article** if the submission is accepted. Please make sure it is accurate and that any funding sources listed in your Funding Information later in the submission form are also declared in your Financial Disclosure statement.<br><br>View published research articles from	The authors have declared that no competing interests exists.

PLOS ONE for specific examples.<br><br>**NO authors have competing interests**<br><br>Enter: *The authors have declared that no competing interests exist.*<br><br>**Authors with competing interests**<br><br>Enter competing interest details beginning with this statement:<br><br>*I have read the journal's policy and the authors of this manuscript have the following competing interests: [insert competing interests here]*<br><br>* typeset	
**Ethics Statement**<br><br>Enter an ethics statement for this submission. This statement is required if the study involved:<br><br>• Human participants<br>• Human specimens or tissue<br>• Vertebrate animals or cephalopods<br>• Vertebrate embryos or tissues<br>• Field research<br><br>Write "N/A" if the submission does not require an ethics statement.<br><br>General guidance is provided below. Consult the submission guidelines for detailed instructions. **Make sure that all information entered here is included in the Methods section of the manuscript.**	Bermuda Hospitals Board, and Bermuda Ethics Committee gave consent for this study to go ahead. COnsent is in written form from both institutions. Please see supporting information.

Powered by Editorial Manager® and ProduXion Manager® from Aries Systems Corporation

**Format for specific study types**<br><br>**Human Subject Research (involving human participants and/or tissue)**<br>• Give the name of the institutional review board or ethics committee that approved the study<br>• Include the approval number and/or a statement indicating approval of this research<br>• Indicate the form of consent obtained (written/oral) or the reason that consent was not obtained (e.g. the data were analyzed anonymously)<br><br>**Animal Research (involving vertebrate animals, embryos or tissues)**<br>• Provide the name of the Institutional Animal Care and Use Committee (IACUC) or other relevant ethics board that reviewed the study protocol, and indicate whether they approved this research or granted a formal waiver of ethical approval<br>• Include an approval number if one was obtained<br>• If the study involved *non-human primates*, add *additional details* about animal welfare and steps taken to ameliorate suffering<br>• If anesthesia, euthanasia, or any kind of animal sacrifice is part of the study, include briefly which substances and/or methods were applied<br><br>**Field Research**<br><br>Include the following details if this study involves the collection of plant, animal, or other materials from a natural setting:<br>• Field permit number<br>• Name of the institution or relevant body that granted permission	
**Data Availability**<br><br>Authors are required to make all data underlying the findings described fully available, without restriction, and from the time of publication. PLOS allows rare exceptions to address legal and ethical concerns. See the PLOS Data Policy and FAQ for detailed information.	Yes - all data are fully available without restriction

A Data Availability Statement describing where the data can be found is required at submission. Your answers to this question constitute the Data Availability Statement and **will be published in the article**, if accepted.<br><br>**Important:** Stating 'data available on request from the author' is not sufficient. If your data are only available upon request, select 'No' for the first question and explain your exceptional situation in the text box.<br><br>Do the authors confirm that all data underlying the findings described in their manuscript are fully available without restriction?	
**Describe where the data may be found in full sentences. If you are copying our sample text, replace any instances of XXX with the appropriate details.**<br><br>• If the data are **held or will be held in a public repository**, include URLs, accession numbers or DOIs. If this information will only be available after acceptance, indicate this by ticking the box below. For example: *All XXX files are available from the XXX database (accession number(s) XXX, XXX.).*<br>• If the data are all contained **within the manuscript and/or Supporting Information files**, enter the following: *All relevant data are within the manuscript and its Supporting Information files.*<br>• If neither of these applies but you are able to provide **details of access elsewhere**, with or without limitations, please do so. For example:<br><br>*Data cannot be shared publicly because of [XXX]. Data are available from the XXX Institutional Data Access / Ethics Committee (contact via XXX) for researchers who meet the criteria for access to confidential data.*<br><br>*The data underlying the results presented in the study are available from (include the name of the third party*	All relevant data are within the manuscript and its Supporting Information files

and contact information or URL).<br>• This text is appropriate if the data are owned by a third party and authors do not have permission to share the data.<br><br>* typeset	
Additional data availability information:	Tick here if your circumstances are not covered by the questions above and you need the journal’s help to make your data available.

Manuscript

Click here to access/download;Manuscript;thesis no figures dec 2022.docx

An exploration of the emotional response among nurses in Bermuda, during the Covid-19 pandemic

Author: Adam Moore (Masters in Public Health, Brunel University)

Contact: writetoadammoore @gmail.com

Advisor: Dr. Navami Leena BDS,MBA,PgCert HE,FHEA,FRSPH,MIHM) Assistant Course Director Public Health Practitioner Apprenticeship at Coventry University, Honorary Lecturer at Brunel University.

Contact: navami.leena @brunel.ac.uk

Abstract of the research:

The Novel Coronavirus Covid-19 has had a huge impact on healthcare systems globally. Nurses spend the most time providing one-to-one care for Covid-19 patients; and are most at risk from contracting the virus (Sethi, 2020). During the course of the Covid-19 pandemic to date; nurses caring for patients within an already undersubscribed workforce have weathered challenging shifts with heavy workloads, and limited equipment (Jackson et al., 2020; International Council of Nurses, 2020). Data from previous respiratory pandemics has shown that nurses suffer from stress and anxiety due to challenges faced inside of work; and stigma in the community, with concern about passing the disease to loved ones exacerbating feelings of isolation (Gao et al., 2020). The Covid-19 pandemic is predicted to bring about further exodus from the workforce; (WHO, 2020) and it is imperative that research be conducted into how to better support nurses, physically and emotionally, so that they avoid burnout, and can continue to provide compassionate care.

This paper demonstrates that nurses working in Bermuda are no exception in terms of feeling stress and anxiety at the outset of the pandemic. This thesis reviews international literature; and investigates the emotional responses of nurses in Bermuda, analysing data from interviews with front-line nurses. It is established that confidence in treating Covid-19 patients has grown as a result of developments in research and availability of the vaccine; and workplace camaraderie between nurses provides a valued support network. Nurses express that they could be better supported by clearer communication of pandemic planning as it evolves; and better access to vital equipment to keep themselves and their patients safe. More research is recommended to better inform interventions aimed at providing improved holistic care for nurses: policies to offer counselling and support; easier access to activities that nurses found beneficial; and pandemic preparedness education.

Author's declaration:

This statement is to certify that, to the best of the author’s knowledge, the content of this thesis is original. All assistance received in preparing this paper, and all contributory sources have been acknowledged.

Acknowledgements:

The author wishes to thank Navami Leena for indispensable guidance throughout the planning, conducting and writing of the thesis. Also, Sammy Bishop, for her assistance with proof-reading, formatting, and super human patience. Thanks also to everyone who participated in interviews, and the wider team at King Edward Memorial Hospital.

Table of Contents

1. Introduction ... 9

1.1 Effect of Covid-19 pandemic on nurses to date ... 9

1.2 Impact of the virus in Bermuda ... 10

1.2.1: Graph showing Covid-19 cases in Bermuda ... 11

1.3 Research questions: Aims and Objectives ... 11

2. Literature Review ... 12

2.1 Nurses' emotional response to respiratory pandemics ... 12

2.2 Search strategy ... 13

2.2.1: PRISMA Chart of Scopus search strategy: ... 13

2.3 Literature Review: Research methods and analysis ... 13

2.4 Discussion of Themes ... 14

Figure 1. Pictorial representation of themes identified in primary research ... 14

3. Research Design and Methodology ... 17

3.1 Methodology ... 17

3.1.1: Gantt Chart of Research ... 4

3.2 Sample and Recruitment ... 4

Table 1. Distribution of sampling among nurse subjects ... 4

3.3 Ethical Issues ... 5

4. Findings ... 6

4.1 Themes identified as an outcome of the analysis of data ... 6

5. Discussion and Analysis: ... 13

5.1 Strengths and Limitations of the study ... 15

6. Conclusions ... 16

7. Recommendations ... 16

8. References ... 18

9. Appendices ... 22

1. Introduction

1.1 Effect of Covid-19 pandemic on nurses to date

The novel coronavirus (Covid-19), first detected in Wuhan, China, in December 2019, has had a devastating impact worldwide. The World Health Organisation states that as of 28th April 2021,there have been148,329,348 confirmed cases reported globally, including 3,128,962 deaths (WHO, 2021). Nurses on the frontline of previous respiratory epidemics, such as Middle East Respiratory Syndrome (MERS) and Severe Acute Respiratory Syndrome (SARS), in 2002 and 2012, respectively, were under increased pressure due tochallenging work environments coupled withstaff shortages(Gao et al., 2020). These factors, combined with fear of transmitting a disease contracted at work; compounded by stigmatisation in the community,lead nurses to experience occupational stress and anxiety, reportedly much higher than other healthcare disciplines(Sethi et al., 2020).Such emotional responses can lead to occupational burnout.Shah et al., (2021) note that burnout and stressful work environment are the most commonly cited reasons for nurses considering leaving the profession. Research into emotional responses of nurses working in the Covid-19 raise similar concerns (Ross, 2020.)

The health and wellbeing of nurses is of paramount importance; and supporting nurses both practically and emotionally is essential to preserving their health, especially in the midst of a global pandemic (Maben and Bridges, 2020).Moreover, prior to the Covid-19 pandemic, the global nursing workforce was at a shortfall of 5.9 million nurses. This coupled with an aging workforce, struggling with increasing workloads and higher incidence of occupational burnout, is the predicted precursor to greater deficits in coming years. Pandemic-related burnout could therefore predict an exodus in experienced nurses (International Council of Nurses, 2020; WHO, 2020; Woo et al., 2020) The safeguarding of nurses' guarantee to work in a safe, supportive environment where concerns are addressed; is essential in order to aid retention and recruitment of new staff.

The above literature highlights that nurses have strong emotional responses to caring for patients during the pandemic, for example, increase of stress and anxiety, which can have a long lasting effect, and can also cause nurses to leave the profession. At present, there is a

lack of research into the experience of nurses working in Bermuda's response to the Covid-19 pandemic.

1.2 Impact of the virus in Bermuda

The global nursing shortage is felt in Bermuda; isolated as it is, in the Atlantic Ocean, with only one acute healthcare centre, King Edward Memorial Hospital (KEMH), serving the population of 62,000. The hospital's nursing staffquota exceeds the country's recently initiated nursing college training capabilities; and approximately 75% of nursing staff come from overseas; from a total of 25 countries (See Appendix A for Diversity Nursing Report). Bermuda saw its first cases on March 23rd 2020; with the initial spike in cases occurring in December 2020, peaking at 253 active cases; after which new infections declined- please see Graph1, below for a timeline. (UN, 2019; Bernews, 2021). Bermuda's isolated geographical location combined with limited bed capacity means that Covid-19 presents a risk to infrastructure being overwhelmed. Hospital Chief of Staff,Wesley Miller recognises that 'Our maximum capacity is not something that could be sustained for very long as our staff would be stretched. This type of burnout has been seen overseas' (Finighan, 2021). Demographically, Bermuda has anaging population,with citizens over 60 anticipated to account for 24.9% of the population by 2026 (Government of Bermuda, 2016). Furthermore, with high density of patients living with chronic health problems, make its residents particularly vulnerable to respiratory illnesses such as covid-19 (UN, 2019; Government of Bermuda, 2016).

1.2.1: Graph showing Covid-19 cases in Bermuda

1.3 Research questions: Aims and Objectives

An explorative study that aims to explore the emotional responses of nurses who treat Covid 19 patients in Bermuda, with the objective of gaining a better understanding of their experience. The researcher aims to answer the following questions with a view to gathering data that can used to inform policy to better support front line nurses during the current, and future pandemics:

-What emotional responses did nurses have and what caused these responses?

-What problems did nurses face at work during the pandemic that lead to an adverse emotional response?

-How has life outside of work changed; and what emotional response is associated with this change?

-What has generated positive emotional responses amongst nurses during the COVID 19 pandemic?

In Bermuda as elsewhere globally, quality nursing care is essential to achieve good patient outcomes. With data from this study, it is hoped that interventions can be developed to support nurses in Bermuda to continue delivering compassionate, effective care; avoid burnout, and retain staff in employment during the ongoing-pandemic and thereafter.

2. Literature Review

2.1 Nurses’ emotional response to respiratory pandemics

The Covid-19 pandemic is an evolving situation, with researchers investing all resources to better understand, treat and eradicate the disease. Indeed, globally, of $421.3 million invested into research during 2020, only 1.8% was not specifically related to Covid-19 (Brown and Head, 2020).With the aim of achieving an understanding of what emotional responses respiratory pandemics have elicited from nurses in the past, the researcher carried out initial searches on CINAHL, MEDLINE and Google Scholar into nurse experiences during the SARS and MERS epidemics. Evidence shows nurses working in respiratory epidemics are likely to become stressed at work; and suffer knock-on effects in home life; due to difficult conditions at work; stigma experienced in the community, and isolation due to self-quarantine due to concern about passing the disease to family members (Gao et al., 2020).

2.2 Search strategy

Initial searches were conducted on 1st March 2021. Boolean operators used:

Nurse AND attitudes AND COVID 19

Results were limited to full-text, academic Journals, published 2019-2021, in English language. Utilising CINAHL yielded 103 matches, the majority of which had no link to full text, with many results not focused on nurse experience. MEDLINE, yielded 39 results after exclusion criteria was applied; but again, full-text articles were hard to locate, and the majority were not relevant to the subject of interest. After consulting with Brunel Library staff, the researcher used the same Boolean operators on Scopus. Please see PRISMA chart of Scopus search strategy, below:

2.2.1: PRISMA Chart of Scopus search strategy:

2.3 Literature Review: Research methods and analysis

The FRAMEWORK approach was used to assess the quality of results; as it is useful to interpret a dataset with particular research questions in mind (Ritchie and Spencer, 1994). Of the 16 studies chosen for the literature review (see Appendix B), 6 were large-scale primary research, utilising online clinical questionnaires to assess respondents' views or emotions. The use of scales such as Depression, Anxiety, and Stress Scale-21 (DASS-21);and the Maslach Burnout Inventory, enable researchers to collate and compare results from one population to another,examining the impact of events or interventions, as numerical scores mean that like can be compared for like. Using online questionnaires with Likert scales ensures a much higher response rate than arranging interviews, for example, which require more time and resources(Kahlkeet al., 2014).

Aside from one international literature review (Fernandez et al., 2020), all other papers reviewed primary data, using semi-structured interviews, allowing for more in-

depthpersonalised answers to questions, and freedom to discuss the matters that are important to participants. While conducting interviews are a useful way to gain an insight into an individuals' point of view; data is not transferable to another population, or even another individual working within the same healthcare facility. Results from these qualitative studies give personal, unique and detailed insight into the participants' emotional response; but are not generalizable; however, similar responses being given by participants within the same facility can give a picture of broad findings within a group.

Wuhan's status as the epicentre of Covid-19 mean that research into nurses' experience in China is over-represented in the sample; with a total of 9 out of 16 studies analysed conducted in the country. Research conducted in Wuhan by Tan et al (2020); Liu et al (2020); Wu et al., (2020); and Zhang et al. (2020) all illuminate the emotional response of increased stress as a result of higher, challenging workloads for nurses during the initial outbreak. Furthermore, Yin and Zeng (2020) applying existence, relatedness and growth need theory to this group, recognising the importance of interpersonal relationships for nurses working with Covid-19 patients. Zhao et al (2020) establishes that emotional response of nurses in Wuhan can be shaped by effective leadership; inclusive leadership style helping to sooth stress and diminish work-based anxiety.

International research from every other continent triangulates findings from China. Aksoy and Kojac (2020) found that nurses in Turkey experienced increase in anxiety; with Altmayer et al. (2021) and Garcia-Martin et al. (2020) presenting supporting data from Paris, France and Andalusia, Spain, respectively. While some studies were focussed on a particular aspect of the nursing role, shift patterns, for example; all data reinforced key themes: nurses' emotional response of stress to higher workload within the workplace; stress due to stigma outside of work, perceived or otherwise; and a positive emotional response of satisfaction at doing job well, and feeling of dedication to the profession despite tough times(Hoseinabadi et al., 2020; Galehdar et al., 2021). The FRAMEWORK analysis model (Ritchie and Spencer, 1994) was used to identify themes within the studies selected. Themes are discussed below.

2.4 Discussion of Themes

Figure 1. Pictorial representation of themes identified

2.4.1 Stress due to challenges at work

All studies found that increased workload as a result of the Covid-19 pandemic lead to increase in stress, and increase in chance of experiencing anxiety and depression. The potential for healthcare workers to suffer from longer term, Post-Traumatic Stress Disorder (PTSD) type illness is identified; and it is established that PTSD is more prevalent among regular staff rather than non-acute or agency nurses; while front-line worker are most likely to suffer burnout (Wu at al., 2020). Nurses discuss the challenges of higher workload meaning that they are not always able to deliver the care they would like to; due to shortages of both time and resources. This 'erosion of care' is seen as affront to their dedication to the role (Galehdar et al., 2020); While each paper reviewed represents nurses as both dedicated to the professional and increasingly stressed; the urgency of work-based support (Zhang at al, 2020); and wider state-based policy to ensure nurses needs are met is highlighted frequently from global data (Fernandez at al., 2020.

2.4.2 Satisfaction in nursing role

Nurses are proud to be working to look after Covid-19 patients, and proud to be an integral part of the pandemic response; this is a positive emotional response to increased workload.Nurses, in general, take pride in their work, and having a sense of duty to work during time of international uncertainty (Wu, Zhao et al. 2020) In Wuhan, there is a willingness to care for Covid-19 patients; which grew when equipped with pandemic-related training and guidance (Zhang et al., 2020). Nurses are consistently shown to be motivated to engage with Covid-19patients in acute environments; but need support, both in work, with role-specific training and education, and also out-of-work, with time to rest, relax, and spend time with loved ones (Nie et al., 2020).

2.4.3 Stigma driven isolation and anxiety

The stigma of being a nurse during the pandemic elicited strong emotional responses, leading to further stress and therefor a reduction in quality of life (Yin and Zeng, 2020). Each one of the 16 studies reports that nurses feel that they cannot socialise; and cannot see friends and family- due to self-regulation because of concern about being vectors; and perceived suspicion of transmitting disease contracted at work to others in the

community. There is a recognition that it is a human need to spend time with loved ones; and understanding that family and private life is connected to workplace life(Tan et al., 2020). New nurses are identified as being in need of particular support, and need for all nurses to have training, support and counselling(Garcia-Martin et al., 2020). These interventions are identified as being the responsibility of national governments and policy makers, but also healthcare facility managers and clinical nurse managers. Academics highlight the need for more research in order to inform policy that was better protect nurses (Nie et al., 2020);

2.4.4 Reliance on clinical leaders to assuage stress

A common thread through the literature was the impact that those within leadership roles could have on the emotional state of others working within the healthcare organisation. Managers with inclusive attitudes were found to quell stress and anxiety by having clear communication with nurses(Zhao et al., 2020). Management teams within the hospital are identified as being in charge of the practical management of patient flow- i.e. addressing bed capacity issues or staff shortages; but also serve as a visible and accessible source of practical and emotional support during stressful times(Garcia-Martin et al., 2020). Managers set the tone of the organisation, and in doing so can alleviate stress and anxiety amongst team members; with an inclusive leadership style facilitating clear communication, both top-down and bottom-up. Fernandez et al (2020) shows that nurses internationally value managers who are responsive to nurse needs, in terms of practical issues- i.e. access to personal protective equipment (PPE), and also emotional support- having a debrief after stressful events, for example.

3. Research Design and Methodology

3.1 Methodology

The researcher uses an ontological approach; finding that the qualitative data reviewed in the literature yields the deepest, most thorough understanding of thoughts and feelings of an emotional nature. Emotional responses are complex and multi-faceted, and cannot easily be defined with tick boxes and scales (Kahke et al., 2014).Therefore, focussed, semi-structured, one-on-one interviews were chosen in order to gain a deep understanding of the emotional responses of nurses.

The overall study design uses a grounded theory approach to data collection and analysis. Please see Gantt chart below (3.1.1) for full chronology of planning,, conducting and analysing data. Structured one-to-one interviews using open ended questioning were conducted, via Zoom. All interviews were carried out between 5th April and 12th April 2021. For interview outline please see Appendix C. Interviews were recorded with the Otter transcription app; reviewed manually for accuracy; and analysed using the FRAMEWORK method (Ritchie and Spencer, 1994). Datawas stored on a hard drive and kept locked in the researchers' home with all files password protected.

3.1.1: Gantt Chart of ResearchSchedule

3.2 Sample and Recruitment

The sample consisted of nurses working at KEMH, Bermuda. The advertisement poster (Appendix D) states that the researcher is interested in attitudes of nurses working with Covid19 patients. The poster was displayed in staff rooms within Acute Departments within the hospital; as well outside the Staff Canteen, and on the hospital intranet.

During the interview process, positive cases were on increasing in Bermuda (See Graph 1, section 1.2). Motivated by the impetus to find willing participants who would find time between busy shifts, convenience sampling was chosen to recruit subjects. Nurses interested were to respond to an email address, and provided with a Participant Information Sheet (PIS) and Consent Form (Appendices E, F, respectively. Please see Table 1, below, for full sample information. After PIS were reviewed, and written consent had been given, email was used to arrange a mutually convenient time to meet for a Zoom interview.

The researcher initially planned to collect data from 16 participants; as this figure meets the aim of research to be small enough to make the data manageable, yet large enough to provide a rich, textured understanding of the experience (Sandelowski, 1995). Time restraints, however, encouraged theresearcher to limit numbers further. It was found that questions had been fully answered and the natural conclusion of the interview was usually around 15 minutes in length, although some were up to 25 minutes. Thematic saturation had been reached at 9 interviews; in that no new ideas or viewpoints were being propagated; Interviews were terminated at this point.

Table 1. Distribution of sampling among nurse subjects

Participant number	Gender	Years of Nursing	Years at BHB	Achievement	Number of Covid patients nursed (approx.)	Country of training
1	M	10	8	Masters	50	Philippines
2	F	11	4	Masters	30	St. Lucia
3	F	11	3	Bachelors	10	Ireland
4	F	42	32	Bachelors	12	England
5	F	13	3	Bachelors	20	Dominica
6	M	7	0.5	Bachelors	300	England
7	F	8	4	Bachelors	10	Ireland
8	F	12	4	Bachelors	5	Jamaica
9	F	3	3	Diploma	10	Bermuda

3.3 Ethical Issues

Strict adherence to local and international ethical guidelines is essential; as the study collects data from human participants. Ensuring the anonymity of participants; and confidentiality of their responses, is paramount, and data must be protected to preserve the identity of those interviewed; as well as individual characteristics of any other patients or staff mentioned within interviews. The particularly sensitive; emotive nature of the matter at hand means it is imperative careful consideration is applied to participant aftercare. The research proposal was reviewed and approved by Brunel Research Ethics Online; by the Bermuda Hospitals Board, and also the Bermuda Ethics (Approval letters can be found in Appendix G, H, and I, respectively.) While some of the participants were known professionally to the researcher, no participant were put under any pressure to take part, and reminded that they could withdraw at any time. Interviews were anonymised, without names assigned, and the audio recordings and transcriptions were treated with the highest confidence

4. <u>Findings</u>

4.1 Themes identified as an outcome of the analysis of data

4.1.1 Initial fear, receding as pandemic develops

All nurses interviewed expressed that the initial emotional response at the outset of the first wave of Covid-19, was one of fear. This caused stress and anxiety, mostly due to unknown nature of symptom effect and transmission. Words such as ‘frightened’, ‘afraid’, ‘wary’, ‘nervous’ and ‘fearful’ were used in relation to initial emotional response; and very much intertwined with feelings of stress and anxiety. Participant 5 (P5) succinctly explains how these emotional responses are inter-related:

‘*Initially there was a lot of fear. We weren’t clear on what PPE we were supposed to be using… I felt afraid because it felt like, you know, you go into work and you might catch Covid and die.’ (P5)*

A recurring theme from participants experiencing fear and anxiety, was concern about transmitting the disease to those close to them, leading to self-imposed exclusion from loved ones, often family residing in the same home. This brought about emotional response of loneliness and feeling of isolation; explored later in the ‘Social stigma’ section. Initial emotional responses of fear and anxiety are quelled by better clinical understanding of how the disease is contracted, and treated, which gave nurses hope, and also provided peace of mind while treating Covid-19 patients. P9 states:

‘Its brand new, so of course you’re going to have a level of anxiety… after understanding a bit more about it, you realise okay, let me do when I needed to do to protect myself, so I will go in and ensure I wear the proper PPE.’

The majority of participants stated their fear, anxiety and stress levels were assuaged by the development and steady roll out of the Covid-19 vaccination programme. P4 discusses emotional response to the third spike in cases, when many staff have been fully vaccinated; explaining that while nurses might feel reassured to be vaccinated; the nature of the disease remains an unpredictable and challenging one to treat:

'I feel like we feel a bit better because were vaccinated, like the staff seem to be a little more confident, but you still have that anxiety...because they're in a room and you can't pop in and out to assess them. So you're looking through a window at them all the time… that still brings up kind of fear, for the patient for the most part this time.'

4.1.2 Anxiety due to lack of knowledge and perceived lack of equipment

All participants' emotional response to initial lack of information about the disease was anxiety and panic. Elevated stress levels were exacerbated by debate about what constitutes appropriate PPE; and also that supplies were insufficient for nurses to carry out their job roles safely. P9 states

'*In the beginning I think I was one of those people who were wary and kind of... unsure… because there was so little research on what proper PPE is and how [the virus] is transmitted. It was just kind of, the unknown, I had a bit of... not anxiety, but just unease in caring for the patients. Now that we know a bit more about it, and how it's transmitted, and the proper PPE for it, it's just like any other patient.'*

Participants universally felt less anxious, and more confident in caring for patients at work, and therefore less stressed outside of work, when they felt they had the correct PPE to protect themselves. Provision of this equipment is regarded as the job of hospital bosses.

Several participants voiced frustration that they were not provided with hand washing facilities outside of patient rooms; meaning they were unable to follow international guidelines about hand hygiene during donning and doffing PPE. P9 states

'*you're being told to do one thing, and it's not being able to be facilitated through the lack of a sink outside of the room'*.

P8 had similar concerns:

'I'm already taking all this stuff off in the room… and when I come outside, I don't only need, I don't only want to sanitise. I want to wash my hands. That's all'.

Whereas other staff felt their needs were met, and that lessons had been learnt during the fluctuation of cases. An example is provided by P1:

'*We asked for better protective gowns… we had this meeting and the manager was involved, HR was involved… because, you know, since we are the Covid floor and we have demands, he had to make some changes… like better PAPRs [Powered Air Purifying Respirators]… we demanded it and we got it'*.

Participants from this department felt encouraged and supported that their concerns were listened to; and that staffing levels were improved on the ward. The addition of tables outside patient rooms that could be used as stations to safely don and doff PPE meant staff felt more comfortable and confident in following guidelines for protecting themselves at work, and in doing so, assuaging emotional response of stress and anxiety about transmitting the disease to their close contacts at home.

4.1.3 Anxiety due to perceived lack of plan

P7 states there is conflict between advice given by clinical managers and shift supervisors on the appropriate PPE and equipment; adding to the anxiety felt by staff that they are not properly protected,

'*Then other departments… all wearing N95s, ICU are all wearing N95s, fully dressed. So just huge conflict'*.

P3 also voices emotional response of concern and frustration at conflict between messages on correct protection:

'Last night we had house cleaners on the floor cleaning rooms in hazmat suits. And we're just making do with tiny little masks and the odd shield when we can get them. Really, I don't know.., it's just like nurses are expected to carry on and manage, it doesn't matter what the situation is.'

Participants also felt there was a lack of communication about preparedness for increasing Covid-19 numbers, particularly before each spike, eliciting emotional response of concern and frustration. Staff interviewed used words such as 'abandoned' (P3) about a lack of communication about the practicalities of bed capacity and management, and perceived lack of 'a plan'. P3 states

‘They may well have a plan but they weren’t voicing it to us, you know so you’re kind of wondering what are they going to do next – you’re worried, because you are the one that’s going to deal with this, you know if there’s a delay in somebody getting an ICU bed, it’s you that has to look after the patient until that point. So, they need to be there to reassure us that there are doing something…. As soon as the Covid comes, you don’t see management as much.’.

Clinical managers and shift supervisors in charge of the hospital at nights and weekends are the link between top level management and floor level staff; and as such, are relied upon to provide support when staff need it.The role of managers and supervisors is not only practical in terms of supplies and staffing; (although these both have an impact on the stress levels of nurses at work) staff also relied on managers for reassurance. P5 discusses a conversation with her clinical manager after a week of surge in positive patients:

‘*I spoke to my manager, and said to her ‘What is the plan? Because I thought, you know the numbers are going up... She said we don’t really have a plan, you know, we just do the same thing that we’ve been doing. .. and really, I would have preferred if we had an optimum plan… when it gets to the point that we can’t take anybody else in, what do we do?’*

4.1.4 Social stigma leads to a feeling of isolation

All participants disclosed that their status as healthcare workers (HCW) made them feel stigmatised within the community, eliciting an emotional response of feelings of isolation and loneliness. While nurses interviewed explained that they had self-imposed isolation measures, out of fear of transmission to friends and family; they also reported decrease in socialisation due to perceived stigma of members of the public, explains P2:

‘I really didn’t want to go anywhere, or be seen to be going anywhere, because you’re a nurse and you’re in the hospital and you don’t want to talk to anybody else, especially those that aren’t working the hospital, because you just don’t. You’re afraid you’re going to give it to them or something.’

This stigma was experienced among friends; P6 states:

‘Even recently I went for a meal with non-nurse friends…and it’s like… you kind of feel, you know, they…I swear they treat you differently. Because they’re worried as well, aren’t they?’

Also in public, with next-door neighbours; P7 speaking about an interaction with their landlady, who lives next door:

'I parked my bike, and then I was waiting to speak to her, and she wouldn't put down the window… and then I realised that she was waiting for me to get off my bike, so she could get out of her car and go into the house.'

4.1.5 Increased cooperation at work; mutual support amongst nurses

All participants had the emotional responses of confidence, courage and felt a morale boost from the mutual support and cooperation between their nursing colleagues. Most nurses reported that their first port of call during troubled times were their colleagues, providing both practical and emotional support when things are difficult, and enjoying a 'shared understanding' of the mentally challenging aspects of the job. P1, working on the initial covid-19cohort ward stated

'Because of the pandemic we became, our floor become more of someone, we were united because we formed… a group…and everyone was just so cooperative and it made us unite…. So I think yeah, having the co-workers support at that time really helped us.'

The closeness exhibited among colleagues, and nurses' reliance on their co-workers for emotional support, as well as practical matters, is a response to the high workload and often upsetting events in working life of acute care nurses in a pandemic:

'We support each other… we have a group chat where we check up on one another and see how we're doing. You know if we have, if we had a bad shift or a busy shift, you know, we kind of like debrief and speak to one another. Just basically support each other, you know somebody afterwards, somebody might call and they might see you cry, you know, you might just need the moments of encouragement, and let them know that they're not in it alone and you're there as well.'

All interviewees expressed gratitude for the emotional support provided by their colleagues during difficult times- the mutual understanding enjoyed by co-workers was repeatedly highlighted as an important factor to the closeness of these relationships. P6 , when asked if any changes could be made to support nurses, stated

‘*People are talking about how [the pandemic] actually affects peoples mental health, the actual things that are stressful…nothing gets done. Nothing. It’s like, the black sheep if it, nothing gets spoken about. We need more communication’.*

Whereas P5 felt her feelings were addressed, stating that as numbers rose during each wave,

‘*I think our managers surely ask us, do you all think you’re ok? Do you feel so stressed you need to speak to somebody that can help*?’

Notably, this nurse was the only respondent that expressed knowledge of the Employee Assistance Programme (EAP) counselling service.

4.1.6 Social interactions and holistic activities outside of work

As discussed, nurses interviewed experienced a level of loneliness and isolation due to their status as healthcare workers. The most oft-cited resource to counter emotional response of these feelings was interactions with loved ones; closely followed by holistic activities- time spent outside; enjoying nature, or exercising. When asked what they could do to make themselves feel better outside of work, P7 state

‘*Spinning. Exercise, basically. Yeah, ‘cos when they gym shut down I had a mental breakdown… that’s kind of the only thing; and I picked up drawing again, and I’ll be doing my gardening. Lonely activities, basically.’*

P9, meanwhile, also expresses that exercise helps calm the stress of emotional response associated with a busy or stressful shift:

‘*Being active, just getting out there and just doing things makes you feel better…Because you know, you get so caught up being tired, and you go home, eat and sleep, and then you just come back to work, and it’s kind of depressing.’*

P2 speaks of their partner, stating that

‘We have curfews and lockdowns, I have company… so that made a difference…, we cook stuff, we go outdoors and go for walks, enjoy nature, pray.’

P3 echoes this sentiment,

'You know just go… forcing yourself to get up and go for a walk or just clear your head a bit. I haven't taken up yoga or meditation... Just having, you know, a good few close colleagues were able to talk it through, definitely helps me, you know'.

Participants with live-in partners valued them highly as a resource for emotional support during times of high stress and anxiety, although several nurses disclosed that it helps them to speak with someone who '*has an understanding and appreciation of what [we]* do' (P3). The majority of participants report that they rely heavily on their colleagues for support outside of work as well as inside; even prior to Covid-19. P5 states that

'Before covid, I'm not much of a social person. So most of my friends are people who work in [my department] so we hung out together… yeah, we support each other.'

While not being able to see friends and family face-to-face; nurses valued the knowledge that their loved ones are healthy. P8 states:

'I think probably for me, just knowing my people are safe. My friends, my coworkers, my family. Everyone is ok, and Covid-free. That is comforting.'

This confidence in health stems in part from the continued global rollout of the vaccine programme. The majority of participants (7/9) stated that their stress and anxiety levels were reduced and they felt more confident at work; and more hopeful for the future both inside and outside of work, due to the vaccine. P4 expressed concern that

'We have a very large nursing population that won't take it, but they are seeing the results of Covid first hand… that bothers me a lot. How do we convince the public?

5. Discussion and Analysis:

The FRAMEWORK analysis tool (Ritchie and Spencer, 1994) was used to identify, organise and collate the themes discussed above. It was found that the emotional responses of nurses in Bermuda were reflected in the literature from elsewhere, globally. Moderate to severe stress, anxiety and depression symptoms in the early stages of the pandemic, were found in a study of more than 3,000 nurses in New Zealand and Australia as evidenced in Hammond et al (2021). While this Bermuda based research does in no way aim to diagnose mental health conditions; the phrases used by participants, 'anxiety', 'stress' and 'panic', illustrate how they were feeling during the early stages of the pandemic. The concern voiced by P5 that they might come to work 'and die' is a reflection of global events with regards to HCW dying as a result of workplace exposure; as of May 2020, a total of 152,888 infections and 1413 deaths were reported amongst healthcare workers worldwide (Bandyopadhyay et al., 2021).

Participants are anxious to ensure that they are able to meet international standards on PPE and hand hygiene; in order to care for patients effectively; and also keep themselves and their families safe. This concern is shared with nursing colleagues in Guangdon Province, China, during the initial outbreak (Nieet al, 2020). Health promotion, and the provision of practical suppliesessential for treatment delivery, is the responsibility of national governments and international partnerships. The Covid-19 pandemic brought about a multiagency approach to service delivery; with partnerships outside of the healthcare sphere; for example, UK-based household appliance manufacturer Dyson producing ventilators for use in hospitals (BBC, 2020). Bermuda, small and isolated as it is, takes some cues from UK healthcare guidelines, and some from USA, resulting in compounding confusion among nurses concerning PPE. A global shortage, and debate concerning best practice with regards to PPE,i.e. utility of surgical masks vs. N95 respirators (Ng et al., 2020); sets the backdrop for anxieties concerning availability of protection to avoid unnecessaryrisk at work. In March 2020 the WHO note that stock shortage is putting nurses at risk; leading planners to question whether it could be advisable to reuse or customize what had been considered one-use-only equipment (WHO 2020a; Sureka et al, 2020). Intersectionality within the procurement and allocation of essential supplies- PPE; testing kits, and later vaccines, meant that the UK was able to provide Bermuda with much needed supplies (Royal Gazette, 2021).

Advances in research, coupled with the utility of newly developed vaccines; means thatnurses in Bermuda feelbetter protected against the virus, and therefore less anxious at work; and

ergo, in homelife. The reliability of vaccination is cited by the majority of nurses as important to assuaging stress around contracting Covid-19. 2 of the 9 nurses interviewed disclosed that they had chosen not to be vaccinated as time of data collection; demonstrating that healthcare professionals are subject to the same anxieties concerning vaccination as the non-healthcare workers. Aksoy and Koçak(2020) interviewed nurses in Turkey about Covid-19related stress at work; establishing that high stress at work is mirrored by high stress at home and with family. The diminution of stress and anxiety that accompanies a better knowledge of the disease is a reflection of the 'rational understanding' leading to a decrease in stress, identified among nurses in Wuhan (Lie et al., 2020).

Emotional response of anxiety felt by participants about lack of cohesive hospital-wide strategy is reflected in data from Spanish Emergency Departments: Garcia-Martin et al (2020) investigated opinions of new nurses dealing with the initial outbreak with similar themes arising, with one nurse stating '*Every day, there are changes and it's a mess. You'll come to work and say, "Where should I go? It is a little bit chaotic what's going on right now due to the current pandemic.*' The rapidly changing procedures and protocols for positive patients in Bermuda is a microcosm of the global situation- everything changes so quickly, so response must be rapid, pragmatic and decisive. Clear communication on the particulars of the changing protocol has proved to be a challenge for healthcare infrastructure in Bermuda, as the rest of the world. The evidence that Bermuda has fared relatively well in dealing with covid-19 cases and vaccination rollout (WHO, 2021) suggests that government and healthcare services did follow an effective plan that has, to date, been relatively successful. If it were possible to keep frontline staff reassured of a plan, and its details, this may have gone some way to put nurses' minds at rest.

Emotional response of loneliness and isolation felt by nurses in Bermuda ,due to stigmatisation by interactions with neighbours, friends, other members of the community at the, is detected elsewhere globally (Zolnikov and Furio, 2020). Feelings of uncertainty, isolation and depression are on the increase for many groups (Vostanis and Bell, 2020). Stressful events at work and feelings of being stigmatised within the community means that nurses are particularly at risk. There are channels for help and counselling within the hospital; for example the EAP counselling programme mentioned by P5; although they could be under-utilised. The strong connection that nurses felt amongst their colleagues is evidence

of how important workplace bonds have become to nurses in Bermuda, for support and a listening ear after stressful events or a difficult shift.

Many guest workers nurses in Bermuda live alone; so do not have the benefit of loved ones nearby, exacerbating loneliness. This sentiment echoes the concerns of nurses interviewed in Wuhan, who expressed the need to see their loved ones and enjoy supportive relationships, which is crucial for dealing with stressful situations in a healthy way (Yin and Zeng 2020), The other resource valued by participants, holistic activities such as spending time outside, and specifically exercise; was limited by the closure of the onsite hospital-subsidised gym. The Staff Wellness programme that encouraged staff who would otherwise not be interested in engaging in exercise was halted, due to concern first about Covid-19 transmission, then potentially needing to use the gymnasium as a staff testing facility. This prolonged closure was a barrier to staff getting exercise that they valued as an effective way to manage their stress and anxiety.

5.1 Strengths and Limitations of the study

The study was limited in scope and sample size, with only interviewed 9 of 374 registered working at KEMH. This represents a snapshot of the participants alone and therefore cannot be generalised. While data saturation suggests that views may be commonly held among nurses working at the facility; the results are not transferable. Some of the participants are known personally to the researcher. While this allowed for free and frank speaking; with participants stating their feelings open and honestly; the Hawthorne Effect could play a part in the data gathered. The researcher is, in addition, an inexperienced interviewer. Some research bias is detectable; which could have skewed results. The evidence that only 2 of 9 participants stated they had not been vaccinated is not a reflection of low vaccination rates of nurses in Bermuda at time of interviews (Strangeways, 2021). It is conceivable that nurses who felt that their views may not have been popular; or were controversial; were less motivated to take part; this potentially increases sampling bias; and limits the generalisability of the findings..

6. Conclusions

The data gathered in this study illuminates the emotional responses of nurses in Bermuda. Initial fear of the virus was overcome by knowledge gained due to emerging research and, and nurses were reassured in their work due to the newly developed vaccine. Anxieties about the correct PPE and its availability; and lack of communication of a cohesive plan to floor staffexacerbate stress responses. Additionally, emotional response of loneliness; feelings of isolation caused by stigma; and barriers to spending time with loved ones contribute to a dissatisfaction at an unfulfilling social and home life. Nurses found solace in support from their colleagues; both inside and outside of work, enjoying the support found in strong bonds between co-workers, with increased cooperation and emotional support. Participants were given succour in time spent with loved ones and knowing they are safe, whilst enjoying holistic activities, to soothe workplace borne stresses.

It is in the interest of the population of Bermuda; as an isolated country, with a disproportionately elderly, unwell population, that nurses' concerns are addressed in order to provide for their needs, and ultimately retain the workforce. The main limitation of the study is the small sample size; with limited generalisability; but as the first study of its kind in Bermuda, results are useful as an exploratory case study, with a view to further research into the views of a wider sample. The emotional response of nurses to workplace-based advertising for the Covid-19 vaccine, and associated uptake, will be a particular area for further research to investigate, as a matter of clinical urgency in the ongoing pandemic response. Some potential workplace policy interventions stem from the study, with the aim of improving the chances of positive emotional response of nurses during the pandemic:

7. Recommendations

At national level, the imposition of policy toimplement support systems and relevant budgeting and service allocation to provide support for nurses during and after pandemics. This could include wellness initiatives, with subsidised gym and exercise memberships; increased availability of optional one-to-one counselling services. Nurses may utilise these services; and find them useful, if they had knowledge about them. Low cost measures such as

adverts on individual institutions’ intranet, or posters in staff rooms could educate nurses about counselling services, for example.

Further research and development into region-specific tailored pandemic preparedness plan and training for nurses. A plan must be established for rollout of pandemic services to aid understanding of decision making and educate floor-level staff about what to anticipate. Education; drills and assessment could provide for smoother transition to pandemic-forward services, to assuage stress level. Feedback must be utilised from recipients to ensure relevance and practicality. This is a challenge due to the quickly evolving situation, yet facilitating new protocol being cascaded to staff in handover huddles/team meetings could be a quick and effective way to keep staff up-to-date about changes, without requiring any further human or financial resources.

Education for all healthcare professionals on the utility of the vaccine as safe is essential, in order that they can make the informed decision to take the vaccine in order to protect themselves and their loved ones; and encourage others to do the same.Further research into the above recommendations would be necessary to gauge the impact on satisfaction; and retention of nurses in the field, and at institutional level

8. References

Aksoy, Y. E., &Koçak, V. (2020). Psychological effects of nurses and midwives due to COVID-19 outbreak: The case of Turkey. Archives of psychiatric nursing, 34(5), 427-433.https://doi.org/10.1016/j.apnu.2020.07.011 [Accessed 28/6/21]

Altmayer, V., Weiss, N., Cao, A., Marois, C., Demeret, S., Rohaut, B., ... & Cadet, E. (2021). Coronavirus disease 2019 crisis in Paris: A differential psychological impact between regular intensive care unit staff members and reinforcement workers. Australian Critical Care, 34(2), 142-145. https://doi.org/10.1016/j.aucc.2020.11.005 [Accessed 29/6/21]

Bandyopadhyay, S., Baticulon, R. E., Kadhum, M., Alser, M., Ojuka, D. K., Badereddin, Y., ... &Khundkar, R. (2020). Infection and mortality of healthcare workers worldwide from COVID-19: a scoping review. medRxiv. https://doi.org/10.1101/2020.06.04.20119594 [Accessed 29/6/21]

Bernews, 2021. Bermuda Covid-19 Cases- Bernews. Taken from Bermuda Covid-19 Test Statistics From March 2020 - Current - Bermuda Covid-19 Coronavirus News - Bernews [Accessed 28/4/21]

British Broadcasting Corporation (24 April 2020) Dyson Covid-19 ventilators are 'no longer required'. Available online at https://www.bbc.com/news/business-52409359 [Accessed 25/6/21]

Brown, R. J., & Head, M. G. (2020). Monitoring investments in coronavirus research and development. The Lancet Microbe, 1(2), e61. https://doi.org/10.1016/S2666-5247(20)30039-2 [Accessed 29/6/21]

Fernandez, R., Lord, H., Halcomb, E., Moxham, L., Middleton, R., Alananzeh, I., & Ellwood, L. (2020). Implications for COVID-19: a systematic review of nurses' experiences of working in acute care hospital settings during a respiratory pandemic. International Journal of Nursing Studies, 103637. https://doi.org/10.1016/j.ijnurstu.2020.103637 [Accessed 28/6/21]

Finnighan, G. (2021, April 16th) Get vaccinated to ease pressure on health staff, urges doctor. The Royal Gazette. Available online at: https://www.royalgazette.com/health/news/article/20210416/get-vaccinated-to-ease-pressure-on-health-staff-urges-doctor/ [Accessed 9/6/21]

Galehdar, N., Toulabi, T., Kamran, A., &Heydari, H. (2021). Exploring nurses' perception of taking care of patients with coronavirus disease (COVID-19): A qualitative study. Nursing Open, 8(1), 171-179. https://doi.org/10.1002/nop2.616 [Accessed 28/6/21]

Gao, X., Jiang, L., Hu, Y., Li, L., & Hou, L. (2020). Nurses' experiences regarding shift patterns in isolation wards during the COVID-19 pandemic in China: A qualitative study. Journal of clinical nursing, 29(21-22), 4270-4280. https://doi-org.ezproxy.brunel.ac.uk/10.1111/jocn.15464 [Accessed 28/6/21]

García-Martín, M., Roman, P., Rodriguez-Arrastia, M., Diaz-Cortes, M. D. M., Soriano-Martin, P. J., & Ropero-Padilla, C. (2020). Novice nurse's transitioning to emergency nurse during COVID-19 pandemic: A qualitative study. Journal of Nursing Management. https://doi-org.ezproxy.brunel.ac.uk/10.1111/jonm.13148 [Accesed 29/6/21]

Government of Bermuda, Department of Statistics (2016) Population and Housing Census, 2016. Available online at: 2016-Census-Report.pdf (bernews.com) [Accessed 29th April 2021)

Government of Bermuda: Department of Statistics (2016) Bermuda's Population Projections 2016-2026. Available online at: https://www.gov.bm/sites/default/files/Bermuda-Population-Projections-2016-2026.pdf [Accessed 9/6/21]

Hammond, N. E., Crowe, L., Abbenbroek, B., Elliott, R., Tian, D. H., Donaldson, L. H., ... & Delaney, A. (2021). Impact of the coronavirus disease 2019 pandemic on critical care healthcare workers' depression, anxiety, and stress levels. Australian Critical Care, 34(2), 146-154. https://doi.org/10.1016/j.aucc.2020.12.004 [Acccessed 28/6/21]

Hoseinabadi, T. S., Kakhki, S., Teimori, G., &Nayyeri, S. (2020). Burnout and its influencing factors between frontline nurses and nurses from other wards during the outbreak of Coronavirus Disease-COVID-19-in Iran. Investigacion y educacion en enfermeria, 38(2). https://doi.org/10.17533/udea.iee.v38n2e03 [Accessed 29/6/21]

INTERNATIONAL COUNCIL OF NURSES POLICY BRIEF (2020) Global Nursing shortage and Nurse Retention. Available online at: https://www.icn.ch/sites/default/files/inline-files/ICN%20Policy%20Brief_Nurse%20Shortage%20and%20Retention.pdf [Accessed 30/04/21]

Jackson, D., Anders, R., Padula, W. V., Daly, J., & Davidson, P. M. (2020). Vulnerability of nurse and physicians with COVID-19: Monitoring and surveillance needed.https://doi.org/10.1111/jocn.15347 [Accessed 29/6/21]

Kahlke, R. M. (2014). Generic qualitative approaches: Pitfalls and benefits of methodological mixology. International Journal of Qualitative Methods, 13(1), 37-52.

Liu Y, Zhao M, Zhuang Q, Xu L, He Q. A comparison of COVID-19, SARS and MERS. PeerJ. 2020 Aug 19;8:e9725. doi: 10.7717/peerj.9725. PMID: 32879801; PMCID: PMC7443081. [Accessed 30th April 2021]

Liu, Y. E., Zhai, Z. C., Han, Y. H., Liu, Y. L., Liu, F. P., &Hu, D. Y. (2020). Experiences of front-line nurses combating coronavirus disease-2019 in China: A qualitative analysis. Public Health Nursing, 37(5), 757-763. https://doi.org/10.1111/phn.12768 [Accessed 28/6/21]

Maben J., & Bridges, J. (2020). Covid-19: Supporting nurses' psychological and mental health. Available online at: https://doi.org/10.1111/jocn.15307 [Accessed 6/2/2021]

Ng, K., Poon, B. H., Kiat Puar, T. H., Shan Quah, J. L., Loh, W. J., Wong, Y. J., ... & Raghuram, J. (2020). COVID-19 and the risk to health care workers: a case report. Annals of internal medicine, 172(11), 766-767.

Nie, A., Su, X., Zhang, S., Guan, W., & Li, J. (2020). Psychological impact of COVID-19 outbreak on frontline nurses: A cross-sectional survey study. Journal of clinical nursing, 29(21-22), 4217-4226. https://doi.org/10.1111/jocn.15454 [Accessed 28/6/21]

Ritchie, J. and Spencer, L. (1994) Qualitative Data Analysis for Applied Policy Research. In: Bryman, A. and Burgess, B., Eds., Analyzing Qualitative Data, Routledge, London.http://dx.doi.org/10.4324/9780203413081_chapter_9 [Accessed 29/6/21]

Ross, J. (2020). The exacerbation of burnout during COVID-19: A major concern for nurse safety. Journal of Perianesthesia Nursing, 35(4), 439-440.

Sandelowski, M. (1995). Sample size in qualitative research. Research in nursing & health, 18(2), 179-183. Available online at: https://doi.org/10.1002/nur.4770180211 [Accessed 25/6/21]

Sethi, B. A., Sethi, A., Ali, S., & Aamir, H. S. (2020). Impact of Coronavirus disease (COVID-19) pandemic on health professionals. Pakistan journal of medical sciences, 36(COVID19-S4), S6–S11. https://doi.org/10.12669/pjms.36.COVID19-S4.2779

Shah, M. K., Gandrakota, N., Cimiotti, J. P., Ghose, N., Moore, M., & Ali, M. K. (2021). Prevalence of and factors associated with nurse burnout in the US. JAMA network open, 4(2), e2036469-e2036469. doi:10.1001/jamanetworkopen.2020.36469 [Accessed 9/6/21]

Strangeways, Sam, for The Royal Gazette (2021) 26 per cent of BHB doctors, 50 per cent of nurses not vaccinated - The Royal Gazette | Bermuda News, Business, Sports, Events, & Community | The Royal Gazette Online, April 21st 2021.

Sureka, B., Sinha, A., Tak, V., Garg, M. K., Bhatia, P. K., Bhardwaj, P., Nag, V. L., Dutt, N., Elhence, A., Bhaskar, S., Bishnoi, A., Chauhan, N., Bishnoi, N. R., &Misra, S. (2020). Customized personal protective equipment (PPE): Solution to conservation and management of supplies during the coronavirus disease 2019 (COVID-19) pandemic. Journal of family medicine and primary care, 9(5), 2180–2182. https://doi.org/10.4103/jfmpc.jfmpc_556_20

Tan, R., Yu, T., Luo, K., Teng, F., Liu, Y., Luo, J., &Hu, D. (2020). Experiences of clinical first-line nurses treating patients with COVID-19: A qualitative study. Journal of nursing management, 28(6), 1381-1390. https://doi-org.ezproxy.brunel.ac.uk/10.1111/jonm.13095 [Accessed 28/6/21]

The Royal Gazette (March 27 2021) 'UK to send 22,000 Covid vaccines to Bermuda' Available online at: https://www.royalgazette.com/health/news/article/20210326/uk-to-send-22000-covid-vaccines-to-bermuda/ [Acced 25/6/21]

United Nations (2019) Department of Economic and Social Affairs. Population Dynamics. World Population Prospects 2019. Available online at: World Population Prospects - Population Division - United Nations [Accessed 29th April 2021)

Vostanis, P., & Bell, C. A. (2020). Counselling and psychotherapy post-COVID-19. Counselling and Psychotherapy Research, 20(3), 389-393. https://doi.org/10.1002/capr.12325 [Accessed 29/6/21]

Woo, T.; Ho, R.; Tang, A.; Tam, W. (2020) Global prevalence of burnout symptoms among nurses: A systematic review and meta-analysis, Journal of Psychiatric Research, Volume 123, 2020, Pages 9-20, https://doi.org/10.1016/j.jpsychires.2019.12.015 [Accessed 30th April 2020]

World Health Organisation (2020) State of the world's nursing 2020: investing in education, jobs and leadership. WHO (Geneva). Online at: https://www.who.int/publications/i/item/9789240007017 [Accessed 30th April 2020]

World Health Organisation (2020a). Geneva. Shortage of personal protective equipment endangering health workers worldwide. Available online at: Shortage of personal protective equipment endangering health workers worldwide (who.int) [Accessed 5/6/21]

World Health Organisation (2021) Geneva.Bermuda: WHO Covid 19 Dashboard. Available online at: Bermuda: WHO Coronavirus Disease (COVID-19) Dashboard With Vaccination Data | WHO Coronavirus (COVID-19) Dashboard With Vaccination Data [Accessed 4/6/21]

World Health Organisation (2021) WHO Coronavirus (Covid 19) Dashboard. Available online at: WHO Coronavirus (COVID-19) Dashboard | WHO Coronavirus (COVID-19) Dashboard With Vaccination Data [Acccessed 29th April 2021).

Wu, B., Zhao, Y., Xu, D., Wang, Y., Niu, N., Zhang, M., .& Meng, A. (2020). Factors associated with nurses' willingness to participate in care of patients with COVID-19: A survey in China. Journal of nursing management, 28(7), 1704-1712. https://doi.org/10.1111/jonm.13126 [Accessed 28/6/21]

Wu, Y., Wang, J., Luo, C., Hu, S., Lin, X., Anderson, A. E., ... & Qian, Y. (2020). A comparison of burnout frequency among oncology physicians and nurses working on the frontline and usual wards during the COVID-19 epidemic in Wuhan, China. Journal of pain and symptom management, 60(1), e60-e65. https://doi.org/10.1016/j.jpainsymman.2020.04.008 [Accessed 29/6/21]

Yin, X., & Zeng, L. (2020). A study on the psychological needs of nurses caring for patients with coronavirus disease 2019 from the perspective of the existence, relatedness, and growth theory. International Journal of Nursing Sciences, 7(2), 157-160. https://doi.org/10.1016/j.ijnss.2020.04.002 [Accessed 29/6/21]

Zhang, Y., Wei, L., Li, H., Pan, Y., Wang, J., Li, Q., ... & Wei, H. (2020). The psychological change process of frontline nurses caring for patients with COVID-19 during its outbreak. Issues in mental health nursing, 41(6), 525-530. https://doi.org/10.1080/01612840.2020.1752865 [Accessed 29/6/21]

Zhao, F., Ahmed, F., & Faraz, N. A. (2020). Caring for the caregiver during COVID-19 outbreak: Does inclusive leadership improve psychological safety and curb psychological distress? A cross-sectional study. International journal of nursing studies, 110, 103725. https://doi.org/10.1016/j.ijnurstu.2020.103725 [Acccessed 28/6/21]

Zolnikov, T. R., &Furio, F. (2020). Stigma on first responders during COVID-19. Stigma and Health, 5(4), 375–379. https://doi.org/10.1037/sah0000270

9. Appendices

Appendices:

Appendix A: Bermuda Hospitals Board Diversity Nursing report

Appendix B. Literature review table

Study title, DOI	Type of research/data collection tools used	Population	Themes identified
1. Impact of the coronavirus disease 2019 pandemic on critical care healthcare workers' depression, anxiety, and stress levels<br><br>https://doi.org/10.1016/j.aucc.2020.12.004	Survey. Anonymous web-based study, of HCW in Australia And New Zealand. Utilised the Depression, Anxiety, and Stress Scale-21 (DASS-21).	3770 complete responses, 3039 (80.6%) were from Australia. A total of 2871 respondents (76.2%) were women; the median age was 41 years. Nurses made up 2269 (60.2%) of respondents, with most (2029 [53.8%]) working in intensive care units.	Between 22 and 29% of respondents reported moderate to extremely severe depression, anxiety, and stress symptoms, with women reporting higher scores than men. Although female gender appears to play a role, modifiable factors also contribute to psychological burden and should be studied further.
2. Exploring nurses' perception of taking care of patients with coronavirus disease (COVID-19): A qualitative study	Qualitative. The data were collected through telephone interviews and analysed based on the Lundman and Graneheim's approach.	13 nurses who were taking care of patients with COVID-19, recruited from Lorestan University of Medical Sciences, Khorramabad Iran	Care erosion, nursing professional growth and necessities. Relevance to clinical practice: Improve the work situation of the nurses during care of COVID-19 patients, which can directly or indirectly improve the quality of care of these patients.

(Galehdar et al., 2020)<br><br>https://doi.org/10.1002/nop2.616			
Nurses' experiences regarding shift patterns in isolation wards during the COVID-19 pandemic in China: A qualitative study https://doi-org.ezproxy.brunel.ac.uk/10.1111/jocn.15464	Qualitative: phenomenological; Methods: Semi-structured interviews were used based on the phenomenological research method;	14 nurses from Chinese hospitals	Focussed on shift organisation; recognising need for necessary to arrange shift patterns scientifically and allocate workforce rationally to optimise nursing workforce allocation, reduce nurses' workload, improve nursing quality and promote physical and mental health among nurses during the COVID-19 pandemic
3. Psychological impact of COVID-19 outbreak on frontline nurses: A cross-sectional survey study(Nie et al., 2020)<br><br>https://doi-org.ezproxy.brunel.ac.uk/10.1111/jocn.15454<br><br>Nie, A., Su, X., Zhang, S., Guan, W., & Li, J. (2020). Psychological impact of COVID-19 outbreak on frontline nurses: A cross-sectional survey study. *Journal of clinical nursing*, *29*(21-22), 4217-4226.	Cross-sectional study. Methods: Frontline nurses complete an online survey by convenience sampling, and the survey included six main sections: the General Health Questionnaire, the Perceived Social Support Scale, the Simplified Coping Style Scale, the Impact of Event Scale-Revised,	263 frontline nurses from Guangdong Province, China	Concern for family, being treated differently, negative coping style and COVID-19-related stress symptom were positive related to psychological distress. Perceived more social support and effective precautionary measures were negatively associated with psychological distress

	socio-demographic, occupation and work history.		
4. Implications for COVID-19: A systematic review of nurses' experiences of working in acute care hospital settings during a respiratory pandemic (Fernandez et al., 2020) https://doi.org/10.1016/j.ijnurstu.2020.103637	Systematic review of literature: A structured search using CINAHL, MEDLINE, EMBASE, PubMed, Google Scholar, Cochrane Library, MedNar, ProQuest and Index to Theses was conducted. Review methods:	Thirteen qualitative studies were included in the review. The experiences of 348 nurses included.	Emphasis on supportive care; dedication. Nurses' require Governments, policy makers and nursing groups to actively engage in supporting nurses, both during and following a pandemic or epidemic. Without this, nurses are likely to experience substantial psychological issues that can lead to burnout and loss from the nursing workforce.
5. Factors associated with nurses' willingness to participate in care of patients with COVID-19: A survey in China (Wu; Zhao et al., 2020) https://doi-org.ezproxy.brunel.ac.uk/10.1111/j	This cross-sectional study used a convenience sampling method that included a demographics questionnaire and the Nurses' Perceived Professional Benefits Questionnaire.	The survey was distributed to 1,787 nurses from 36 hospitals in China. Results: In total, 1,176 questionnaires were usable for this research	The vast majority of nurses were willing to participate in care of patients with COVID-19 in China. Surgical nurses and nurses with positive professional perceptions are more likely to be willing to participate in treatment. raising the awareness of infectious diseases and increased pre-disaster training during infectious diseases is critical

onm.13126			
6. Caring for the caregiver during COVID-19 outbreak: Does inclusive leadership improve psychological safety and curb psychological distress? A cross-sectional study (Zhao et al., 2020)<br><br>https://doi.org/10.1016/j.ijnurstu.2020.103725	Design: Cross-Sectional Study with Temporal Separation Settings and Participants: Online questtionare	The researchers recruited 451 on-duty registered nurses from 5 hospitals providing patient care during the highly infectious phase of COVID-19 in January 2020 in Wuhan city.	Recurring or prolonged experiences of stress and anxiety at the workplace, without a mechanism to counter such effects, can culminate into psychological distress. Inclusive leadership style can serve as such a mechanism to curb psychological distress for healthcare workers by creating a psychologically safe environment
7. Psychological effects of nurses and midwives due to COVID-19 outbreak: The case of Turkey (Aksoy and Kocak, 2020) https://doi.org/10.1016/j.apnu.2020.07.011	Questionnaire: Personal Information Form, State-Trait Anxiety Inventory and Intolerance of Uncertainty Scale were used as data collection tools	758 total HCWs: 56.9% of nurses and 43.1% of midwives (Recruited from one HC facility in Turkey)	Nurses and midwives are more likely to suffer from anxiety during Covid outbreak .<br>It was determined that there was a difference between scale scores and difficulties in work, family and private life due to COVID-19
8. Experiences of clinical first-line nurses	Qualitative interviews	Thirty nurses were selected for the study using a	Negative psychological experiences of clinical first-line work and difficulties faced during hands on work. Identification of

treating patients with COVID-19: A qualitative study (Tan et al., 2020) https://doi-org.ezproxy.brunel.ac.uk/10.1111/jonm.13095		purposive sampling method. Data were collected by semi-structured interviews and analysed using content analysis.	need for further support, education, training, counselling.
9. Experiences of front-line nurses combating coronavirus disease-2019 in China: A qualitative analysis (Liu et al., 2020) https://doi-org.ezproxy.brunel.ac.uk/10.1111/phn.12768	Qualitative analysis	Fifteen front-line nurses caring for COVID-19 patients were recruited from two hospitals in Wuhan, China from January 26 to February 5, 2020. Data were collected through semi-structured individual interviews and analyzed using standard qualitative methods.	Facing tremendous new challenges and danger"; "Strong pressure because of fear of infection, exhaustion by heavy workloads and stress of nursing seriously ill COVID-19 patients"; "Strong sense of duty and identity as a healthcare provider"; "Rational understanding of the epidemic'', need for further training
10. A Comparison of Burnout Frequency Among Oncology Physicians and Nurses Working on the Frontline	Questionnaire, 49 questions, 220 medical staff members from the COVID-19 FL and UWs, with a ratio of 1:1.	220 nurses and Doctors working in one oncology unit in Wuhan, China,	Staff working on 'usual wards' s for uninfected patients, medical staff working on the COVID-19 rather than frontline wards had a lower frequency of burnout. Recognition that it is not just frontline staff affected by pandemic- normal operating systems are put on hold for entire HC system .

and Usual Wards During the COVID-19 Epidemic in Wuhan, China (Wu et al., 2020)<br><br>https://doi.org/10.1016/j.jpainsymman.2020.04.008	General information, and the Maslach Burnout Inventory—medical personnel, were gathered		
11. A study on the psychological needs of nurses caring for patients with coronavirus disease 2019 from the perspective of the existence, relatedness, and growth theory (Yin and Zeng, 2020) https://doi.org/10.1016/j.ijnss.2020.04.002	Qualitative, indepth interviews	10 nurses working with COvid patients in Wuhan, China.	Existence, relatedness, and growth needs coexist in clinical nurses.- i.e. need to see friends and family face to face as well as have support systems within work environment.<br><br>The existence needs were mainly reflected in health and security needs, whereas the relatedness needs consisting mainly of interpersonal needs, humanistic concern needs, and family needs; further, the growth needs consisting mainly of knowledge needs.
12. Coronavirus disease 2019 crisis in Paris: A differential psychological impact between regular	Self assessment questionnaires. Anxiety and Depression Scale, the Post-traumatic	Results: Sixty-nine ICU HCWs completed the survey (37 from the team of regular staff members, i.e., from the public health service,	Prevalence of anxiety, depression, and post-traumatic stress disorder symptoms was high. Regular staff members appeared to be more at risk from suffering from PTSD-like symptoms than ‘reinforcement’ or additional staff members.

intensive care unit staff members and reinforcement workers (Altmayer et al., 2021) https://doi.org/10.1016/j.aucc.2020.11.005	Stress Disorder Checklist for DSM-5, McGill Quality of Life Questionnaire-Revised, and 10-item Connor-Davidson Resilience Scale	and 32 from a reinforcement pool, either from non-ICU public health service or from private healthcare interim employment agencies), from 16-bed neurological ICU at La Pitié-Salpêtrière Hospital in Paris, France, which was converted to a COVID ICU.	
13. Novice nurse's transitioning to emergency nurse during COVID-19 pandemic: A qualitative study (Garcia-Martin et al., 2020) https://doi-org.ezproxy.brunel.ac.uk/10.1111/jonm.13148	Qualitative: Semi structured interviews.	Sixteen semi-structured interviews were conducted in sampling at EDs in three different hospitals in Almeria (Andalusia, Spain)	(a) Fears and concerns- i.e. stress; anxiety; infecting a family member<br><br>(b) Organisational issues- lack of planning/bed space; over crowding<br><br>(c) Support for novice nurses: need improved communication, better support for less experienced colleagues
15Burnout and its influencing factors between frontline nurses and nurses from other wards during the outbreak of Coronavirus Disease-COVID-	Cross-sectional study makes comparison between two groups of nurses including	245 Nurses; (response rate 92%). According to hospital policies, frontline nurses	The burnout level in frontline nurses was higher than other nurses, the most important influencing factor was the job stress.

19-in Iran (Hoseinabadi et al., 2020) https://doi.org/10.17533/udea.iee.v38n2e03	frontline (exposure group) and other nurses working in usual wards (non-exposure group) in TorbatHeydariyeh city, Iran.	as an exposure group have to work 12 hour-rotating shifts for one week and have a week off, while other nurses as a non-exposure group entered in the study have the same working condition as before	
16.Psychological Change Process of Frontline Nurses Caring for Patients with COVID-19 during Its Outbreak (Zhang et al., 2020) https://doi.org/10.1080/01612840.2020.1752865	Qualitative descriptive study; semi-stuctured interviews	23 nurses from Covid wards in Wuhan, China	Stress, anxiety, burnout, emotional exhaustion- transitioning to energy renewal; need for work related support.

Appendix C. Outline of Interview Questions

Interview plan sample questions

Questions will be used as starting points for general discussion about attitudes to nursing patients with Covid19. As interviews progress, the program is flexible, and will allow for an organic discussion to develop, with plenty of opportunity for participants to speak freely about the issues they found to be pertinent when caring for Covid patients.

How many patients have you looked after with Covid-19, or possible Covid-19?

How has looking after these patients made you feel?

Has your life changed outside of work because of your status as a nurse? If so, how?

What has helped you during the pandemic so far? Both inside, and outside of work?

Have any changes been made at work to support you while looking after Covid patients?

Would you have liked anything else to be done? Or if there was to be another wave, would you want anything done differently?

Where do you get your information about Covid 19?

Have you heard things about Covid 19 that you don’t believe? Where did you hear them?

How do you feel about the Covid 19 vaccine(s)?

Is there anything else you want to add?

Appendix E. Participant Information Sheet

College of Health, Medicine and Life Sciences
Department of Health Sciences

PARTICIPANT INFORMATION SHEET

Study title
Research question: What are the attitudes and concerns of nurses caring for patients with Covid-19 in Bermuda?

Invitation Paragraph

You have been invited to participate in this study investigating nurse attitudes and concerns about caring for patients with Covid-19. Please see Information below, and get in touch if you want to take part, or have any further questions. My contact details are at the bottom of the sheet.

What is the purpose of the study?

It is hoped that this research will help us to better understand the experience of nurses caring for patients with the virus; in the hope that hospital management will be able to provide tailored support to nurses and other Healthcare professions during this time.
This study is a pre-requisite for the completion on the Masters in Public Health course at Brunel University.

Why have I been invited to participate?
You are invited to participate in this study because you are a Registered Nurse working as Bermuda Hospitals Board, and have likely looked after patients with Covid-19. This study is interested in what you have to say.

Do I have to take part?
No. Participation is entirely voluntary. You do not have to take part, and if you decide you would like to participate, you can withdraw at any time with no further questions asked. If you decide to take part, you will be given this information sheet, and will be asked to sign a consent form. Each participant has the right to withdraw at any time; and you are at liberty to refuse to answer a question or questions without any consequence at all. If you decide to partipate, you are still free to withdraw your data, without giving any reason, until 14 days after your interview has taken plac

What will happen to me if I take part?
The researcher, Adam Moore, will get in touch to arrange a mutually convenient time to ask some questions about your attitudes to caring for Covid positive patients. This will be over Zoom, Skype, or a phone call. The interview will take 30 minutes at the most. Interviews will be audio taped in order that I can listen back and analyse them afterwards.

Are there any lifestyle restrictions?
There are no lifestyle restrictions to this study.

What are the possible disadvantages and risks of taking part?
As covid-19 patients are often very sick; it is anticipated that some information that participants wish to share may be upsetting. Please be aware that information shared will be treated with upmost confidence; and not traceable back to an individual. If content shared is highlights risk to patients, the researcher has a duty to inform Quality and Risk.

What are the possible benefits of taking part?
This study presents an exciting opportunity for nurses to share how they feel about nursing patients with Covid 19. This pandemic has changed so many things about daily life globally, and is a hot topic, not just in healthcare terms, but also socially, politically, economically. Nurses have been at the forefront of direct patient care during these challenging times, and a more detailed understanding of the pressures they face, and their overall experience dealing with Covid19, will be of great interest to all in the healthcare field, and useful for healthcare planners in the future. This is an opportunity to have your voice heard.

What if something goes wrong?
Participants can withdraw at any time. If participants become distressed or upset during interviews, the interview will be terminated. There will be signposting to support services- information on debrief sheet. There will also be a 'cooling off' period. After data has been collected, participants may decide that they do not want it included in the study; and may contact the researcher in the 14 days following- any such request will be honoured.

Will my taking part in this study be kept confidential?
Yes, data will be completely confidential. Names will not be taken, so there is no way that anything a participant states in an interview could be attributable to them

Will I be recorded, and how will the recording be used?
Everything you say will be completely confidential. Individual participants' names will not be recorded; there is no way that anything you say will be attributable to you. Signed Consent forms will be kept in a sealed file, separate to all other data. Recorded data will be analysed by the researcher, and findings will be submitted in the form of a thesis to Brunel University exams board on completion towards the end of 2021.

What will happen to the results of the research study?
Results will be coded (for anonymity) and analysed by the researcher before being reported. The results will be written into a research report, the final piece of work for a Masters' in Public Health, and may later to reported in a scientific journal. In addition, the report will be made available to Bermuda Hospitals Board of Directors. The anonymised research may be

shared with other researchers for further analysis; but at no point will uniquely identifiable data be shared. If you choose to take part in this research, you can obtain a copy of the publication from the researcher.

Who is organising and funding the research?
Research is organised and funded by Adam Moore, Registered Nurse working on Catlin Lindo Ward, as part of a Masters in Public Health, studying remotely from Brunel University, London.

What are the indemnity arrangements?
Brunel University London provides appropriate insurance cover for research which has received ethical approval.

Who has reviewed the study?
This study has been reviewed by the College of Health, Medicine and Life Sciences Research Ethics Committee; and also by Bermuda Ethics Committee.

Research Integrity
Brunel University London is committed to compliance with the Universities UK Research Integrity Concordat. You are entitled to expect the highest level of integrity from the researchers during the course of this research

Contact for further information and complaints:
For general information
Researcher name: Adam Moore.
Email: 1948143@brunel.ac.uk
University Supervisor: Dr.SathyanarayananDoraiswamy
Email: Sathyanarayanan.Doraiswamy@brunel.ac.uk

1.2.1 Graph showing Covid-19 cases in Bermuda

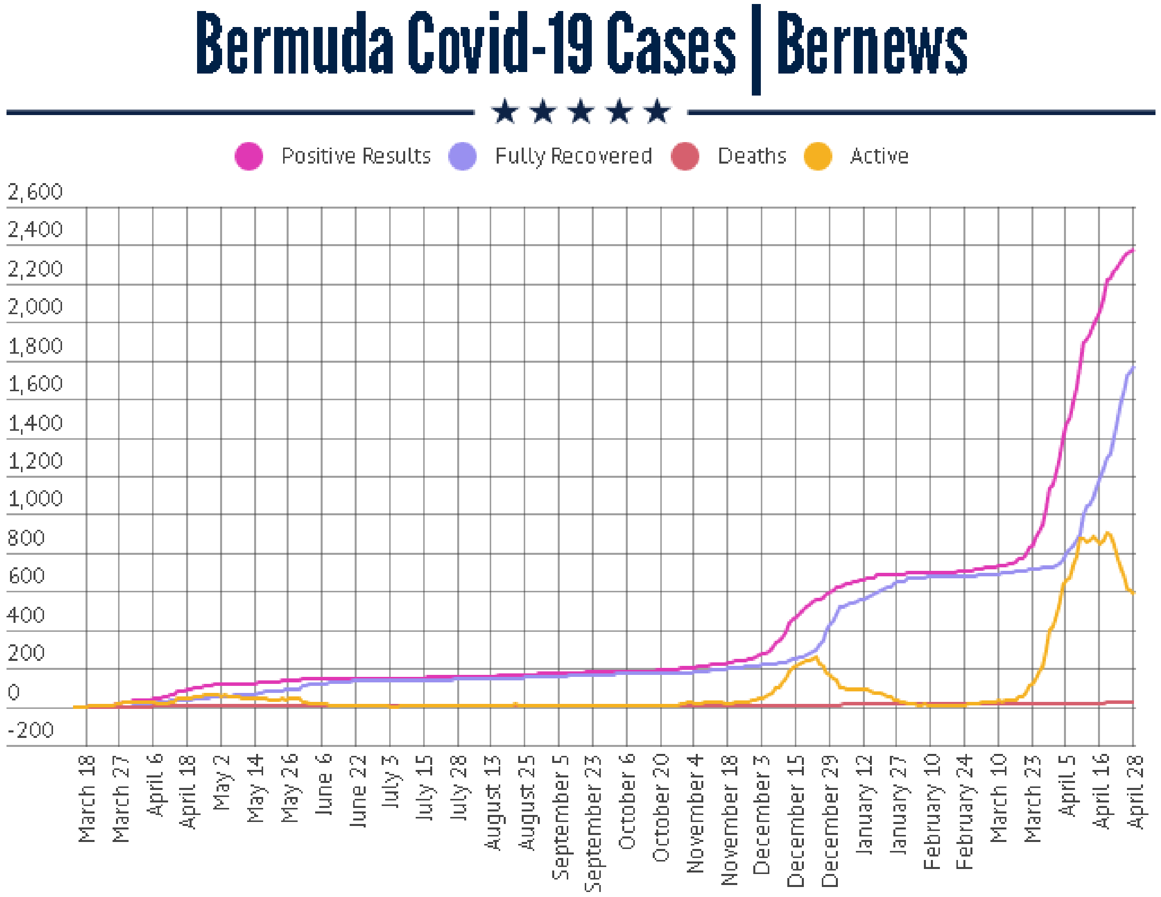

2.2.1: PRISMA Chart of Scopus search strategy:

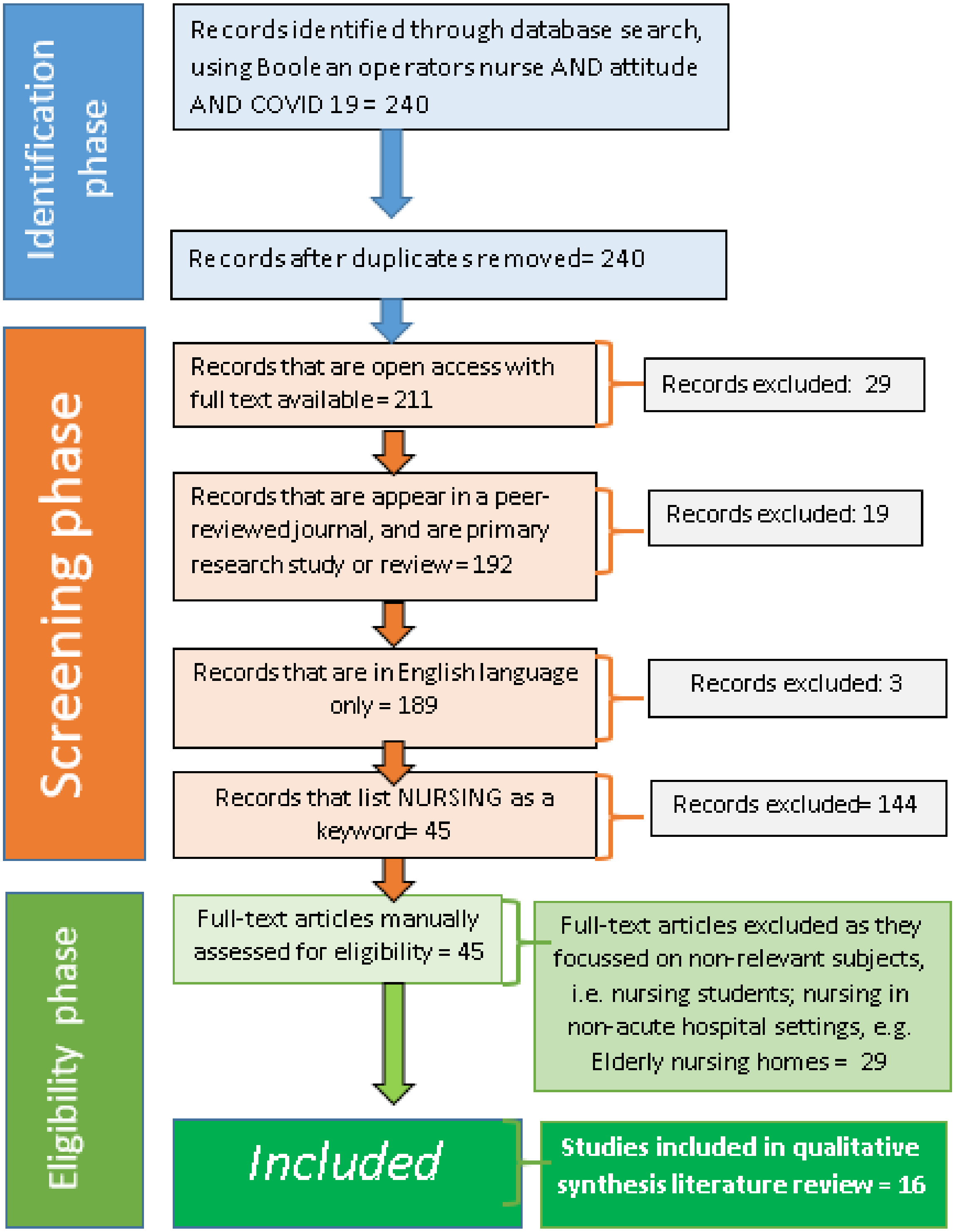
Identification phase
Records identified through database search, using Boolean operators nurse AND attitude AND COVID 19 = 240
Records after duplicates removed= 240
Screening phase
Records that are open access with full text available = 211
Records excluded: 29
Records that are appear in a peer-reviewed journal, and are primary research study or review = 192
Records excluded: 19
Records that are in English language only = 189
Records excluded: 3
Records that list NURSING as a keyword= 45
Records excluded= 144
Eligibility phase
Full-text articles manually assessed for eligibility = 45
Full-text articles excluded as they focussed on non-relevant subjects, i.e. nursing students; nursing in non-acute hospital settings, e.g. Elderly nursing homes = 29
Included
Studies included in qualitative synthesis literature review = 16

Figure 1. Pictorial representation of themes identified

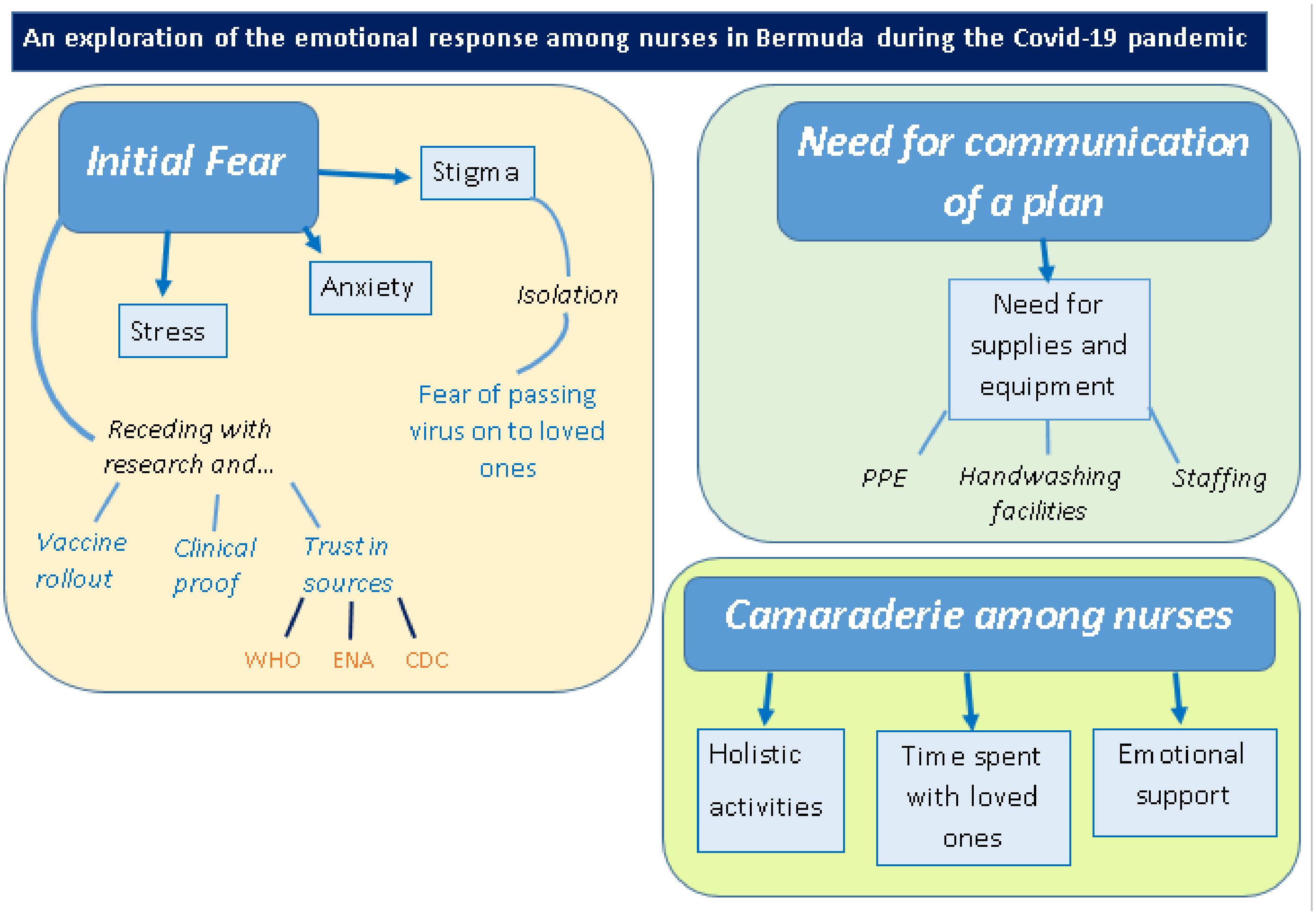

3.1.1: Gantt Chart of Research Schedule

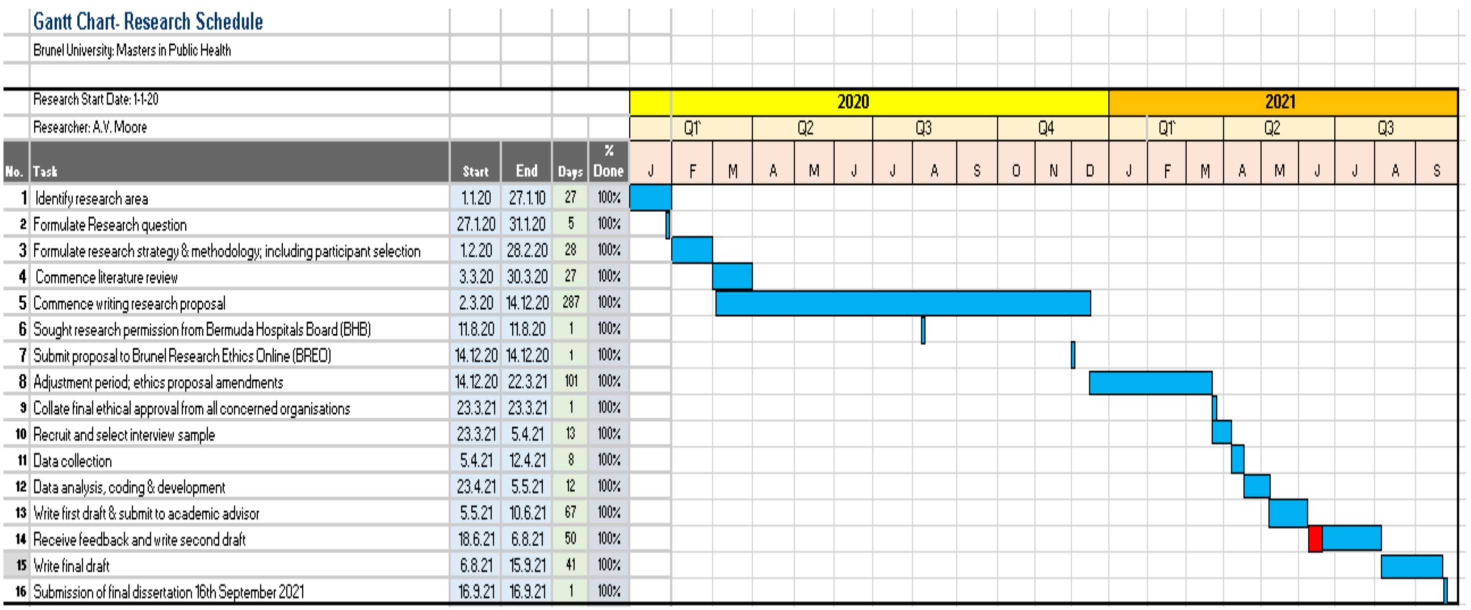

Gantt Chart- Research Schedule

Brunel University: Masters in Public Health

Research Start Date: 1-1-20

Researcher: A.V. Moore

No.	Task	Start	End	Days	% Done
1	Identify research area	1.1.20	27.1.10	27	100%
2	Formulate Research question	27.1.20	31.1.20	5	100%
3	Formulate research strategy & methodology; including participant selection	1.2.20	28.2.20	28	100%
4	Commence literature review	3.3.20	30.3.20	27	100%
5	Commence writing research proposal	2.3.20	14.12.20	287	100%
6	Sought research permission from Bermuda Hospitals Board (BHB)	11.8.20	11.8.20	1	100%
7	Submit proposal to Brunel Research Ethics Online (BREO)	14.12.20	14.12.20	1	100%
8	Adjustment period; ethics proposal amendments	14.12.20	22.3.21	101	100%
9	Collate final ethical approval from all concerned organisations	23.3.21	23.3.21	1	100%
10	Recruit and select interview sample	23.3.21	5.4.21	13	100%
11	Data collection	5.4.21	12.4.21	8	100%
12	Data analysis, coding & development	23.4.21	5.5.21	12	100%
13	Write first draft & submit to academic advisor	5.5.21	10.6.21	67	100%
14	Receive feedback and write second draft	18.6.21	6.8.21	50	100%
15	Write final draft	6.8.21	15.9.21	41	100%
16	Submission of final dissertation 16th September 2021	16.9.21	16.9.21	1	100%

Appendix D. Recruitment poster for Research study

Attention Registered Nurses!

Have you cared for a patient with Covid-19?

Would you be willing to talk about your experience?

A new study into the attitudes and concerns of RNs looking after Covid-19 patients is beginning next month. We need you!

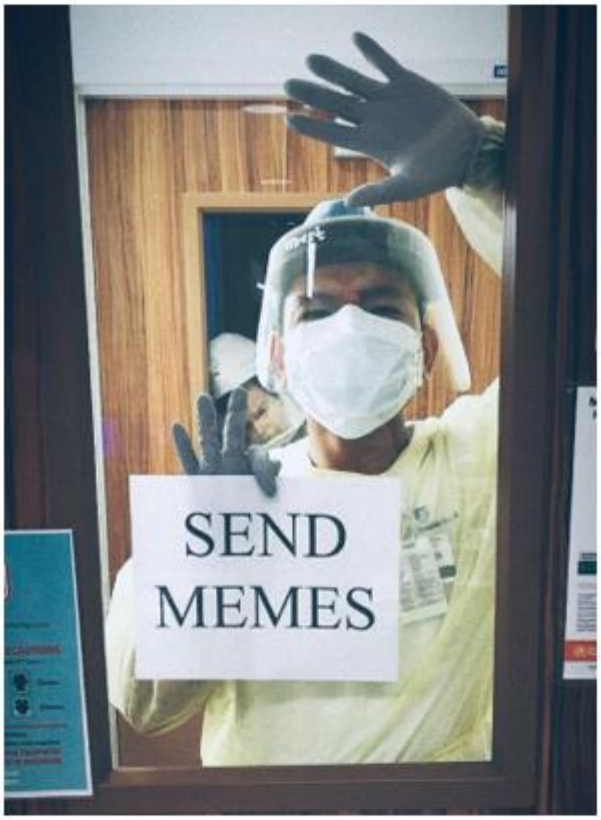

Please contact: 1948143@brunel.ac.uk if you are interested in participating.

It is hoped that the results will help plan support for RNs in the future- your help would be much appreciated.

Please get in touch if you are interested or want to know more.

Please note that participation is totally optional- you can withdraw at any time; and all information disclosed is completely confidential- names will not be taken.

This study is part of a Master's course at Brunel University, London, and has been approved by the College of Health, Medicine and Life Sciences Research Ethics Committee; as well as the Bermuda Ethics Committee. Proposed dates of data collection: 26/02/21-30/4/21

Diversity Nursing Report		
**Country Represented**	**Position**	**Total**
AUSTRALIA	*PATHOLOGY NURSE*	1
	REGISTERED NURSE	5
BAHAMAS	*REGISTERED NURSE*	2
BARBADOS	*E/N CLIN ADMIN MNGR.*	1
	REGISTERED NURSE	23
CANADA	*REGISTERED NURSE*	12
DOMINICA	*REGISTERED NURSE*	1
DOMINICAN REPUBLIC	*REGISTERED NURSE*	1
FIJI	*REGISTERED NURSE*	6
GERMANY	*REGISTERED NURSE*	1
GHANA	*CLIN PSYCH TRI NURSE*	1
GRENADA	*REGISTERED NURSE*	4
GUYANA	*REGISTERED NURSE*	16
INDIA	*REGISTERED NURSE*	19
IRELAND	*COMM PSYCH. NURSE*	1
	REGISTERED NURSE	3
JAMAICA	*CLIN RESOURCE NURSE*	1
	COMM PSYCH. NURSE	1
	REGISTERED NURSE	74
KENYA	*REGISTERED NURSE*	2
NEW ZEALAND	*REGISTERED NURSE*	8
NIGERIA	*REGISTERED NURSE*	2
PHILIPPINES	*REGISTERED NURSE*	49
PORTUGAL	*REGISTERED NURSE*	1
SOUTH AFRICA	*CASUAL R.N.*	1
	REGISTERED NURSE	1
ST. LUCIA	*D.I. NURSE*	1
	REGISTERED NURSE	27
ST. VINCENT	*CLINICAL EDUCATOR*	1
	REGISTERED NURSE	22
TRINIDAD & TOBAGO	*CLINICAL EDUCATOR*	1
	COMM PSYCH. NURSE	1
	REGISTERED NURSE	10
UNITED KINGDOM	*COMM PSYCH. NURSE*	4
	D.I. NURSE	1
	E/N CLIN ADMIN MNGR.	1
	MENTAL WELFARE OFF	1
	REGISTERED NURSE	13
UNITED STATES	*CAS D.I.RESOURCE NUR*	1
	CLINICAL EDUCATOR	1
	REGISTERED NURSE	1
**Grand Total**		**323**

Appendix A: Bermuda Hospitals Board Diversity Nursing report

Click here to access/download
Supporting Information
supporting information.docx
