# Supplementary material for: An exploration of the emotional response among nurses in Bermuda, during the Covid-19 pandemic
Source: PLoS One. 2024 Sep 17;19(9):e0279792. doi: 10.1371/journal.pone.0279792 (PMC11407671; doi:10.1371/journal.pone.0279792)
Supplement: Attachment — Submitted filename: PONE-D-22-34212.pdf [file pone.0279792.s008.pdf]

An Exploration of the Emotional Response among Nurses in Bermuda, during the Covid-19 Pandemic

Summary of the Study

This was an explorative study that aims to explore the emotional responses of nurses who treat Covid-19 patients in Bermuda, with the objective of gaining a better understanding of their experience. They interviewed 9 nurses to get in-depth analysis and exploration of these emerging phenomena. They made also an extensive literature review about previous researches conducted during COVID-19 pandemic. Six themes were elicited and explored to shed the light on these phenomena at Bermuda. They found that there is an initial fear of virus that was overcomed by knowledge. Anxieties about the correct PPE and its availability; and lack of communication of a cohesive plan to frontline staff exacerbate stress responses. Additionally, emotional response of loneliness; feelings of isolation caused by stigma; and barriers to spending time with loved ones contribute to a dissatisfaction at an unfulfilling social and home life. Nurses found solace in support from their colleagues; both inside and outside of work, enjoying the support found in strong bonds between co-workers, with increased cooperation and emotional support. Participants were given succor in time spent with loved ones and knowing they are safe, whilst enjoying holistic activities, to soothe workplace borne stresses.

Strength Points

Study topic is interesting for the international reader; it is about very important phenomena that whole world was encountered and still complaining from its outcomes. The whole article is perfectly written. I feel easy to understand their work, the article was very consistent with itself. Finally, the study has a good implication for the clinical practice, education, and research.

Weak Points

None

Title, Abstract and Key Words

Title is easy to understand. Abstract is very informative. Key Words are good. The aim of work and the research questions were clear and addressed well.

An Exploration of the Emotional Response among Nurses in Bermuda, during the Covid-19 Pandemic

Introduction and Background

It was focused, comprehensive with excellent flow of ideas. The aim of work and the research hypothesis were clear and addressed well. There was a good referencing for each idea. ***I think that this section could be a single paper rather than the whole manuscript*****.**

Materials and Methods

The authors illustrate the method section clearly. A good sampling technique was also used. They presented method and sampling process in a form of flow diagram. They consider the ethics of research conduction.

Statistics

They used appropriate strategy for analysis correctly.

Discussion and Conclusion

They interpret the results and explains what they represent for research and real-life application. They also mentioned the limitations and strengths of the study which is sound and outlined in a good manner. Also, limitations of the study provide good ideas for future research.

References

They were relevant, indexed and referenced correctly.
