# Supplementary material for: An exploration of the emotional response among nurses in Bermuda, during the Covid-19 pandemic
Source: PLoS One. 2024 Sep 17;19(9):e0279792. doi: 10.1371/journal.pone.0279792 (PMC11407671; doi:10.1371/journal.pone.0279792)
Supplement: Attachment — Submitted filename: RESPONSE TO REVIEWERS.docx [file pone.0279792.s009.docx]

RESPONSE TO REVIEWERS

Firstly, thanks much for reviewing this paper. I appreciate your expertise and patience. Below I listed changes to the paper as suggested, responding to your comments. Please do let me know if you have any further feedback. This is the first paper I´ve written for publication, so any guidance at all is much appreciated.

I have edited the study design to be more clear, and the objectives to be more focused.

Please see below for specific changes. Aside from Edits to language, a couple of missed spacebar hits, and a capital letter here and there, I have updated the pre-2016 references with more recent, relevant studies. References can be found at the bottom of this document.

Thank again.

Adam

(Page Number listed correspond to manuscript draft originally submitted)

p. 8 ABSTRACT- fully rewritten with aim, methods, conclusions and recommendations, as suggested. I am aware that my abstract is quite lengthy in comparison to most research paper, but felt it was pertinent to address the findings of the literature review as well as the primary research conducted.

p. 14 LITERATURE REVIEW and OBJECTIVES- I have delineated between the 2 sections of the paper clearly to make it more clear. I outline that a lit review will be conducted to get a picture of the international situation, and then primary research to assess the experience of nurses in Bermuda. As such, there are a few structural changes that I have made throughout the paper. Objective questions have been separated as suggested.

p.16 Edited by ensure that the reader understands this is a lit review of qualitative research papers, followed by conduction of qualitative research

P20. Methodology- distinguished between lit review and quality research

p. 21 Emotional response operational has been properly defined and qualified. Additional references from relevant literature have been used (pleasen see below)

p23. ETHICS: Clarified motivations for ethical research, and type of ethical approvals granted. I also edited to add a brief discussion of measures to ensure validity, reliability, with reference to relevant literature.

**New References:**

Arnetz, J. E., Goetz, C. M., Arnetz, B. B., & Arble, E. (2020). Nurse reports of stressful situations during the COVID-19 pandemic: qualitative analysis of survey responses. *International journal of environmental research and public health*, *17*(21), 8126. [Accessed 20/02/2023]

Chew, N. W., Lee, G. K., Tan, B. Y., Jing, M., Goh, Y., Ngiam, N. J., ... & Sharma, V. K. (2020). A multinational, multicentre study on the psychological outcomes and associated physical symptoms amongst healthcare workers during COVID-19 outbreak. *Brain, behavior, and immunity*, *88*, 559-565. [Accessed 06/02/2023]

Cypress, Brigitte S. EdD, RN, CCRN. Rigor or Reliability and Validity in Qualitative Research: Perspectives, Strategies, Reconceptualization, and Recommendations. Dimensions of Critical Care Nursing 36(4):p 253-263, 7/8 2017. | DOI: 10.1097/DCC.0000000000000253 [Acccessed 09/02/2023]

Hackett, A., & Strickland, K. (2018). Using the framework approach to analyse qualitative data: a worked example. *Nurse researcher*, *26*(2). DOI:[10.7748/nr.2018.e1580](http://dx.doi.org/10.7748/nr.2018.e1580) [Accessed 06/02/23]

Huang, L., Lei, W., Xu, F., Liu, H., & Yu, L. (2020). Emotional responses and coping strategies in nurses and nursing students during Covid-19 outbreak: A comparative study. *PloS one*, *15*(8), e0237303. [Accessed 20/02/23]

Kählke, F., Berger, T., Schulz, A., Baumeister, H., Berking, M., Cuijpers, P., ... & Ebert, D. D. (2019). Efficacy and cost-effectiveness of an unguided, internet-based self-help intervention for social anxiety disorder in university students: protocol of a randomized controlled trial. *BMC psychiatry*, *19*(1), 1-12. [Accessed 10/02/2023]

Khosravani, M., Abedi, H., Rafiei, F., & Rahzani, K. (2017). The association between conscience understanding and clinical performance among nurses working at education hospital of Arak. *Annals of Tropical Medicine and Public Health*, *10*(6). [Accessed 10/02/2023]

Lak, S., Zahedi, S., Davodabady, F., & Khosravani, M. (2018). Conscience understanding among nurses working at education hospital of Arak. *Revista Latinoamericana de Hipertensión*, *13*(3), 246-250. [Accessed 10/02/2023]

Missouridou, E. (2017). Secondary posttraumatic stress and nurses' emotional responses to patient's trauma. *Journal of Trauma Nursing*, *24*(2), 110-115. [Accessed 20/02/2023]

Rose, Jeff & Johnson, C.W (2020) Contextualizing reliability and validity in qualitative research: toward more rigorous and trustworthy qualitative social science in leisure research, Journal of Leisure Research, 51:4, 432-451, DOI: [10.1080/00222216.2020.1722042](https://doi.org/10.1080/00222216.2020.1722042) [Acccessed 09/02/2023]
